# Hypoxia-Induced Modulation of Apoptosis and BCL-2 Family Proteins in Different Cancer Cell Types

**DOI:** 10.1371/journal.pone.0047519

**Published:** 2012-11-05

**Authors:** Audrey Sermeus, Marie Genin, Amélie Maincent, Maude Fransolet, Annick Notte, Lionel Leclere, Hélène Riquier, Thierry Arnould, Carine Michiels

**Affiliations:** Laboratory of Biochemistry and Cellular Biology (URBC), NARILIS, University of Namur – FUNDP, Belgium; University of Edinburgh, United Kingdom

## Abstract

Hypoxia plays an important role in the resistance of tumour cells to chemotherapy. However, the exact mechanisms underlying this process are not well understood. Moreover, according to the cell lines, hypoxia differently influences cell death. The study of the effects of hypoxia on the apoptosis induced by 5 chemotherapeutic drugs in 7 cancer cell types showed that hypoxia generally inhibited the drug-induced apoptosis. In most cases, the effect of hypoxia was the same for all the drugs in one cell type. The expression profile of 93 genes involved in apoptosis as well as the protein level of BCL-2 family proteins were then investigated. In HepG2 cells that are strongly protected against cell death by hypoxia, hypoxia decreased the abundance of nearly all the pro-apoptotic BCL-2 family proteins while none of them are decreased in A549 cells that are not protected against cell death by hypoxia. In HepG2 cells, hypoxia decreased NOXA and BAD abundance and modified the electrophoretic mobility of BIM_EL_. BIM and NOXA are important mediators of etoposide-induced cell death in HepG2 cells and the hypoxia-induced modification of these proteins abundance or post-translational modifications partly account for chemoresistance. Finally, the modulation of the abundance and/or of the post-translational modifications of most proteins of the BCL-2 family by hypoxia involves p53-dependent and –independent pathways and is cell type-dependent. A better understanding of these cell-to-cell variations is crucial in order to overcome hypoxia-induced resistance and to ameliorate cancer therapy.

## Introduction

Cancer is one of the main causes of death in developed countries. Treatments often include the use of chemotherapies but drug resistance is a common cause of treatment failure and relapse. Most chemotherapeutic drugs induce cell death by triggering apoptosis. Apoptosis is regulated by proteins of the BCL-2 (B-cell lymphoma-2) family that mainly act at the mitochondrial level. This family of proteins can be subdivided into three categories according to the function and structure of the proteins [1]. First, the BAX-like proteins, such as BAX (BCL-2 associated × protein) and BAK (BCL-2 homologous antagonist/killer), are death factors. Once activated, they oligomerise at the mitochondria and induce mitochondrial outer membrane permeabilisation, which provokes cell death. The BCL-2-like proteins, such as BCL-2, BCL-xL (B-cell lymphoma-extra large), BCL-w and MCL-1 (myeloid cell leukaemia 1), are survival factors. They form heterodimers with BAX/BAK and inhibit their action. Finally, the BH3 (BCL-2 homology domain 3)-only proteins, such as BID (BH3-interacting domain death agonist), BAD (BCL-2-antagonist of cell death), BIM (BCL-2-interacting mediator of cell death), BIK (BCL-2 interacting killer), PUMA (p53 upregulated modulator of apoptosis) and NOXA, induce apoptosis by neutralizing the antiapoptotic BCL-2-like molecules and, for some of them, by directly activating BAX/BAK. In healthy cells, BH3-only proteins are either not present or kept inactive or sequestered. Following proapoptotic signals, they become transcriptionally upregulated and/or post-translationally modified (and/or relocalised) to gain their full proapoptotic function [1], [2].

One of the main causes of resistance to chemotherapy-induced apoptosis is p53 mutation. p53 is indeed a central player for the induction of apoptosis in stressed cells [3], [4] Another well-known cause of resistance is tumour hypoxia [5]. The effects of hypoxia on cancer cells are cell type dependent and differ according to the intensity and the duration of the lack of O_2_. Severe and prolonged hypoxia may initiate apoptosis or necrosis while mild hypoxia is protective [6], [7]. Indeed, mild hypoxia interferes with several components of the apoptotic pathway at the transcriptional as well as at the post-translational level.

Previous results from our group showed that hypoxia protects tumour cells from apoptosis induced by chemotherapeutic agents. Hypoxia protects HepG2 cells against etoposide-induced apoptosis as well as MDA-MB231 cells against paclitaxel-induced apoptosis [8], [9], [10]. However, in other cell types, hypoxia may have no effect or even aggravate the apoptosis induced by chemotherapeutic agents. It is for example the case for etoposide-induced apoptosis of A549 and MCF-7 cells as well as for epirubicin-induced apoptosis of MDA-MB231 cells [8], [10]. These results have also underlined that cell responses to hypoxia is very complex, involving different signal transduction pathways as well as several transcription factors. Notably, the p53 transcription factor seems to play an important role in the cell behaviour under hypoxia [8]. The aim of this work is to understand how hypoxia may differently influence the chemotherapeutic drug-induced apoptosis in cancer cells. Therefore, we first extended our previous observations using 7 cancer cell lines, originating from various organs and having different p53 status, exposed to 5 apoptosis-inducing drugs used in chemotherapy that target different cellular pathways. In most cases, hypoxia partly prevented cell death and the effect of hypoxia was the same for all the drugs in one cell type. Secondly, we observed that hypoxia modulated the abundance of most proteins of the BCL-2 family in a cell type-dependent manner. Thirdly, in HepG2 cells that are protected by hypoxia against etoposide-induced cell death, we observed a decrease in BAD and NOXA protein level as well as a change in the electrophoretic mobility of BIM_EL_ (BIM extra long) in hypoxia-incubated cells. Results from invalidation studies suggest that the combined loss of BIM and NOXA during hypoxia at least in part accounted for chemoresistance.

## Materials and Methods

### Cell Culture and Hypoxia Incubation

Human hepatoma HepG2 cells, human lung carcinoma A549 cells, human breast adenocarcinoma MDA-MB231 cells, human hepatoma Hep3B cells, human osteogenic sarcoma U2OS cells, human colon adenocarcinoma HT-29 cells and human prostate adenocarcinoma PC-3 cells (ATCC) were maintained in culture in 75-cm^2^ polystyrene flasks (Costar) with respectively D-MEM (low glucose) (Gibco), MEM (Gibco), RPMI 1640 (Gibco), RPMI 1640 (Gibco) containing 50 U/ml penicillin and 50 µg/ml streptomycin (Gibco), Mc Coy’s 5A medium (Gibco), RPMI 1640 (Gibco) and F12 Kaighn’s medium (Gibco) containing 10% of FCS and incubated under an atmosphere containing 5% CO_2_.

For hypoxia experiments, cells were incubated in serum-free CO_2_-independent medium (Invitrogen) supplemented with 0.5 mM L-glutamine (Sigma) with or without etoposide (Sigma), methotrexate (Sigma), paclitaxel (Molecular Probes), cisplatin (Sigma) or camptothecin (Sigma) for 16 hours. The incubation under hypoxia was performed in home-made incubators containing 99% N_2_ and 1% O_2_. The level of hypoxia in the medium in our experimental conditions is 10 mm Hg of dissolved oxygen within the medium. This level of hypoxia is achieved after about 10 min of incubation. PO_2_ level was measured using a dissolved oxygen meter (Inolab Oxi 730) equipped with a Cellox 325 probe. Normoxic control cells were incubated in the same conditions but in normal atmosphere (21% O_2_).

### siRNA Transfection

siGENOME SMARTpool human (Dharmacon; used at 50 nM) BCL2L11 (BIM, M-004383-02), PMAIP1 (NOXA, M-005275-03), BAD (M-003870-02) or TP53 (M-003329-03) were transfected with the DharmaFECT1 transfection reagent (Dharmacon; used at a 1∶500 dilution). When combining BIM and NOXA siRNA, each siRNA was used at 25 nM. The siGENOME RISC-Free Control siRNA (D-001220-01, Dharmacon; used at 50 nM) or siGENOME Non-Targeting siRNA Pool #1 (D-001206-13, Dharmacon; used at 50 nM) were used as negative controls. Cells were incubated for 24 hours with transfection medium and the medium was then replaced by fresh medium with 10% of FCS. 6 hours later (or 30 hours later for BIM siRNA), cells were incubated under hypoxia or normoxia with chemotherapeutic agents or not.

### Subcellular Fractionation

Cells, seeded in 75-cm^2^ flasks, were harvested in saccharose M/4 and homogenized by 3 strokes of a B plunger in a Dounce. The homogenate was centrifuged 10 minutes at 1,418 *g* (Labofuge 400R Heraeus Instruments). The supernatant was recovered and centrifuged at 81,085 *g* (Beckman L7-35, rotor 50 Ti) for a duration dependent of the volume. The pellet was resuspended in saccharose M/4 and named the MLP fraction. The supernatant was concentrated by centrifugation in Amicon Ultra-4 tubes (Millipore) and named the S fraction. All the samples were finally homogenized by sonication.

### Western Blot Analysis

Protein extraction was performed as described in [9]. Proteins were separated by SDS-PAGE on 15% polyacrylamide gel, on 4–12% NuPAGE Bis-Tris gels with MES buffer (Invitrogen) or on 3–8% NuPAGE Tris-Acetate gels (Invitrogen) and then transferred to a PVDF membrane (Hybond-P, Amersham for chemiluminescence detection or Immobilon-FL, Millipore for fluorescence detection). Chemiluminescence detection was performed as described in [11] using overnight incubation with specific primary antibodies. For fluorescence detection, membranes were blocked 1 hour at room temperature in Odyssey blocking buffer (LI-COR) and then overnight at 4°C in Odyssey blocking buffer with 0.1% Tween containing a specific antibody. After 4×5 minutes washes in PBS-Tween 0.1%, the incubation with the secondary antibody (goat anti-rabbit or anti-mouse IRDye-labeled antibody, LI-COR, 1∶10,000 dilution) was performed for 1 hour in Odyssey blocking buffer with 0.1% Tween followed by 4 washes of 5 minutes in PBS-Tween and 2 washes in PBS. The membrane was then dried 1 hour at 37°C and scanned using Odyssey infrared imaging system (LI-COR).

### Immunofluorescence Staining

HepG2 cells were seeded at 40,000 cells/well on glass cover slides in 24-well plates. After incubation with or without etoposide or paclitaxel, immunofluorescence staining was performed as described in [11]. Primary antibody for alpha-tubulin staining was mouse anti-alpha-tubulin (# T5186 Sigma) (1/100 dilution). Alexa Fluor-647-conjugated anti-mouse IgG antibody (Molecular Probes) was used at 1/1,000 dilution.

### Caspase-3/7 Activity Assay

The fluorogenic substrate Ac-DEVD-AFC (BD Pharmingen) was used to measure caspase-3/7 activity according to [12]. Cell extracts were prepared as described in [13]. Cells were seeded in 25-cm^2^ flasks. The assay for caspase-3/7 activity was performed according to [8] using 20 µg of extracts.

### Lactate Dehydrogenase Release Assay

Lactate dehydrogenase (LDH) release was measured with the “Cytotoxicity Detection Kit” from Roche Applied Science as described in [9]. The culture media from incubated cells were removed and centrifuged to pellet the cell fragments and apoptotic bodies. In order to lyse the cells, Triton X100 (Merck) at 10% in PBS was added on this pellet as well as on the cells remaining attached on the bottom of the wells. The percentage LDH release was calculated as follows: [LDH activity in medium (1) + LDH activity of cells fragments and apoptotic bodies (2)]/[(1) + (2) + LDH activity of cells remaining in the wells].

### Transient Transfection and Luciferase Assay

Cell transfection was performed in 24-well plates (50,000 cells per well for HepG2 and MDA-MB231 cells; 30,000 for A549 cells; and 40,000 for Hep3B cells) with SuperFect reagent (Qiagen). 923 ng (1385 ng for MDA-MB231 cells) of the reporter plasmid pGL3-(PGK-HRE6)-tk-luc, which contains 6 HRE cis-elements from the PGK gene linked to the thymidine kinase basal promoter and to the firefly luciferase gene [14], were co-transfected with 577 ng (115 ng for MDA-MB231 cells) of normalization vector (pCMVβ vector coding for the β-galactosidase, Clontech) in medium without serum for 6 hours (or 3 hours for Hep3B cells before replacing the medium with fresh medium). Afterwards, cells were incubated under hypoxia or normoxia for 16 hours. After incubation, cells were lysed in Passive Lysis Buffer (Promega) and β-galactosidase was assayed in parallel to the firefly luciferase activity, assayed using the Luciferase Assay System (Promega).

### Taqman Low Density Array

After the incubation, total RNA was extracted using TRI Reagent Solution (Applied Biosystems). mRNA contained in 2 µg total RNA was reverse transcribed using the “High Capacity cDNA Reverse Transcription kit” from Applied Biosystems according to the manufacturer’s instructions. 100 ng of retrotranscribed total RNA in 50 µl were then mixed with 50 µl of the “Taqman Universal PCR master Mix” (Applied Biosystems) and loaded into one of the 8 fill ports of the microfluidic array. “TLDA Human Apoptosis Panel” are 384-well microfluidic cards that contain assays for 96 human genes. They enable to perform 96 real-time PCR reactions simultaneously for 4 samples. mRNA expression level was quantified using the threshold cycle method with 18S as the reference gene.

### RT Real-time-PCR for mRNA Quantification

After the incubation, total RNA was extracted using TRI Reagent Solution (Applied Biosystems). mRNA contained in 2 µg total RNA was reverse transcribed using SuperScript II Reverse Transcriptase (Invitrogen). Forward and reverse primers sequences are available in Table S1 and were designed using the Primer Express 1.5 software (Applied Biosystem). Amplification reaction assays contained 1× SYBRGreen PCR Master Mix (Applied Biosystem) and primers (Applied Biosystems, 300 nM). RPL13A was used as the reference gene for normalization and mRNA expression level was quantified using the threshold cycle method.

### Heatmap and Statistical Analyses

Heatmaps were generated with the data transformed in logarithm base 2 of the results for TDLA (n = 1) and of the means of three values for qRT-PCR results. Statistical analyses were carried out with ANOVA 1 and Tukey’s multiple comparison tests using GraphPad Prism.

## Results

### Hypoxia has Different Effects on Drug-induced Cell Death in Different Cell Types

In order to obtain further insight of the mechanisms initiated by hypoxia that lead to cancer cell resistance, we extended our previous observations using other cancer cell lines originating from various organs and having different p53 status and other apoptosis-inducing chemotherapeutic drugs that target different cellular pathways. The cell lines used are HepG2 (p53 wild-type hepatoma cells), A549 (p53 wild-type lung carcinoma cells), U2-OS (p53 wild-type osteogenic sarcoma cells), MDA-MB231 (p53 mutant breast adenocarcinoma cells), HT-29 (p53 mutant colon adenocarcinoma cells), Hep3B (p53 null hepatoma cells), and PC-3 (p53 null prostate adenocarcinoma cells) cells. The chemotherapeutic agents chosen are etoposide (a topoisomerase II inhibitor), methotrexate (a DNA and RNA synthesis inhibitor), paclitaxel (an inhibitor of microtubule dynamics), cisplatin (an alkylating agent) and camptothecin (a topoisomerase I inhibitor).

**Figure 1:**
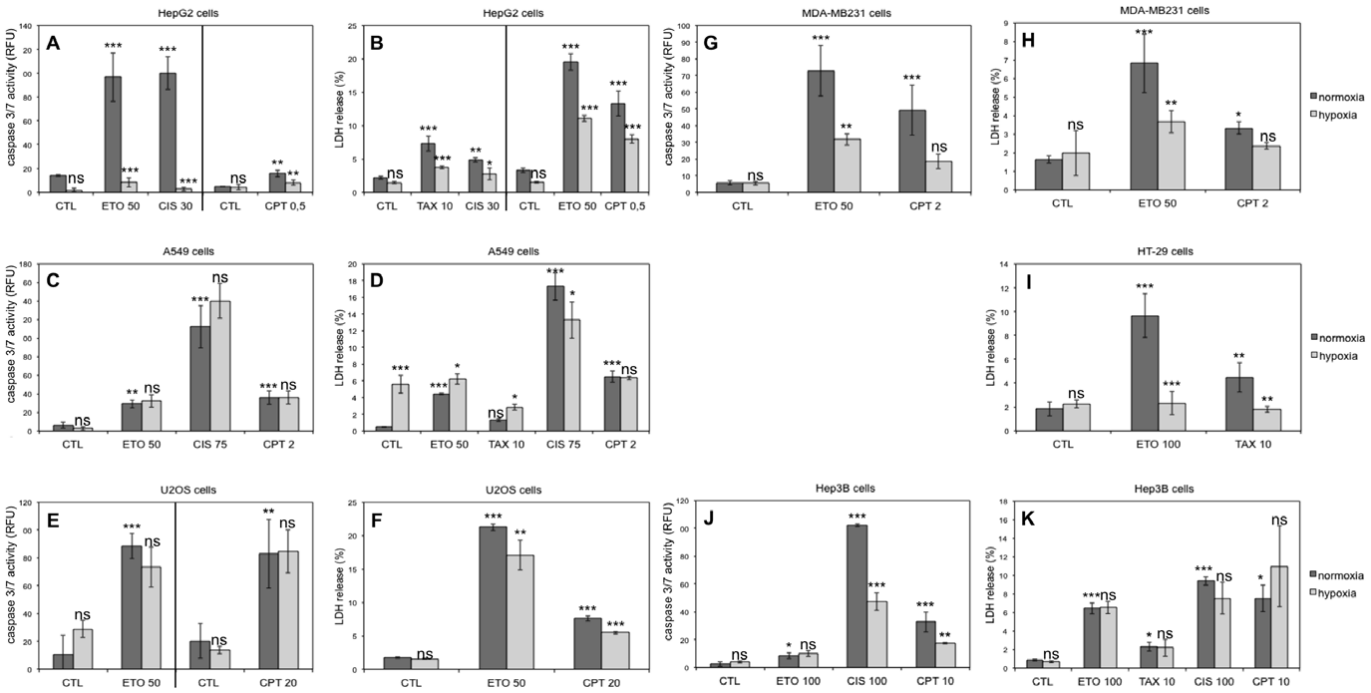
Effect of hypoxia on chemotherapeutic agents-induced cell death. HepG2, A549, U2OS, MDA-MB231, HT-29, Hep3B and PC-3 cells were incubated under normoxia (21% O_2_) or hypoxia (1% O_2_) in the absence (CTL) or presence of etoposide (ETO), paclitaxel (TAX), cisplatin (CIS) or camptothecin (CPT) for 16 (A, C, E, G, J) or 40 hours (B, D, F, H, I, K). The concentration used for each molecule is indicated in the graphs (in µM). (A, C, E, G, J) Caspase-3/7 activity was assayed by measuring the fluorescence of free AFC released from the cleavage of the caspase-3/7 specific substrate Ac-DEVD-AFC. Results are expressed in relative fluorescent units (RFU) as means ±1 SD (n  = 3). (B, D, F, H, I, K) LDH release was assessed. Results are presented in percentages as means ±1 SD (n  = 3). (A-K) Statistical analysis was carried out with ANOVA 1. ns: non-significant; *: P<0.05; **: P<0.01; ***: P<0.001. Symbols above a normoxic column are a comparison with the normoxic control and symbols above a hypoxic column are a comparison with the corresponding normoxic condition (with the same drug).

First, the concentration of drug to use on each cell type was chosen by assaying caspase-3/7 activity after 16 hours incubation with the drugs. In each case, a concentration inducing moderate apoptosis was chosen. For the agents inducing no caspase activity, their overall toxicity was evaluated by measuring lactate dehydrogenase (LDH) release 40 hours after incubation with the agents. Once the concentrations were chosen, the effect of hypoxia (1% O_2_) on the cell death induced by each agent in each cell type was monitored by the two same techniques (Figure 1).

We observed that, in general, the p53 mutated or null cells are more difficult to kill than the p53 wild-type cells. In most cases, the effect of hypoxia on cell death was the same for all molecules in one cell type. However this effect varied according to the cell type. We observed also that hypoxia had more often a protective effect against cell death than an aggravating effect. For example, hypoxia protected HepG2 and MDA-MB231 cells against the cell death induced by all the molecules tested while we observed no effect or an aggravation of drug-induced cell death by hypoxia in A549 and Hep3B cells. Hypoxia can therefore protect p53 wild-type as well as p53 mutated cells. Altogether, these results generated a matrix of data about the effect of hypoxia on the apoptosis induced by 5 molecules in 7 cell types (Table 1). The results showed that the effect of hypoxia is different according to the cell type but constant for all the drugs on one cell type. To the best of our knowledge, this observation has never been made before.

**Table 1:** Effect of hypoxia on chemotherapeutic agents-induced cell death.

cell type	p53 status	assay	**etoposide**	**methotrexate**	**paclitaxel**	**cisplatin**	**camptothecin**
**HepG2**	wt	Ap	50 µM	no death at 10 µM	no death at 5 µM	30 µM	0,5 µM
		CD	50 µM	no death at 50 µM	10 µM	30 µM	0,5 µM
**A549**	wt	Ap	*50 µM*	no death at 10 µM	no death at 5 µM	*75 µM*	*2 µM*
		CD	**50 µM**	no death at 50 µM	**weak death at 10 µM**	*75 µM*	*2 µM*
**U2OS**	wt	Ap	50 µM	no death at 20 µM	no death at 10 µM	weak death at 100 µM	*20 µM*
		CD	50 µM	weak death at 50 µM	no death at 10 µM	no death at 100 µM	20 µM
**MDA-MB231**	R 281 K	Ap	50 µM	no death at 50 µM	no death at 5 µM	no death at 100 µM	2 µM
		CD	50 µM	no death at 50 µM	weak death at 10 µM	no death at 100 µM	2 µM
**HT-29**	R 273 H	Ap	no death at 150 µM	no death at 50 µM	no death at 5 µM	no death at 100 µM	no death at 10 µM
		CD	100 µM	no death at 50 µM	10 µM	weak death at 100 µM	no death at 20 µM
**Hep3B**	null	Ap	*100 µM*	no death at 50 µM	no death at 5 µM	100 µM	10 µM
		CD	*100 µM*	no death at 50 µM	*10 µM*	*100 µM*	*10 µM*
**PC-3**	null	Ap	no death at 150 µM	no death at 50 µM	no death at 5 µM	no death at 100 µM	no death at 10 µM
		CD	no death at 50 µM	no death at 50 µM	no death at 5 µM	no death at 100 µM	no death at 10 µM

HepG2, A549, U2OS, MDA-MB231, HT-29, Hep3B and PC-3 cells were incubated under normoxia (N, 21% O_2_) or hypoxia (H, 1% O_2_) in the absence (control) or presence of etoposide, methotrexate, paclitaxel, cisplatin or camptothecin. Apoptosis was assayed by measuring caspase-3/7 activity (Ap) and overall cell death was assessed by measuring LDH release (CD) after 16 or 40 hour incubation respectively. Caspase-3/7 activity was assayed by measuring the fluorescence of free AFC released from the cleavage of the caspase-3/7 specific substrate Ac-DEVD-AFC. The p53 status of each cell line is specified in the second column: wild type (wt), mutated or null. The concentrations used for each molecule on each cell type are indicated. “weak death” means that some cell death (statistically different from the control) is sometimes observed but not reproducible. A light grey cell indicates that hypoxia decreases drug-induced cell death; a dark grey cell indicates that hypoxia does not modify drug-induced cell death; and a black cell indicates that hypoxia aggravates drug-induced cell death. hypoxia protect against cell death; *hypoxia has no effect on cell death*; **hypoxia aggravates cell death.**

### Expression Profile of mRNA and Proteins Involved in Apoptosis in Different Cell Types

In order to understand why hypoxia protected some cell lines against drug-induced death but not others, we selected four cell lines harbouring different p53 status and for which the effect of hypoxia is different: HepG2, A549, MDA-MB231 and Hep3B cells. We used etoposide as cell death inducer since it is powerful in all four cell lines and its mode of action is well known. The effect of hypoxia on the paclitaxel-induced death of HepG2 cells was also studied.

### Etoposide and Paclitaxel are Active and HIF-1 is Activated Under Hypoxia in all Cell Types

**Figure 2:**
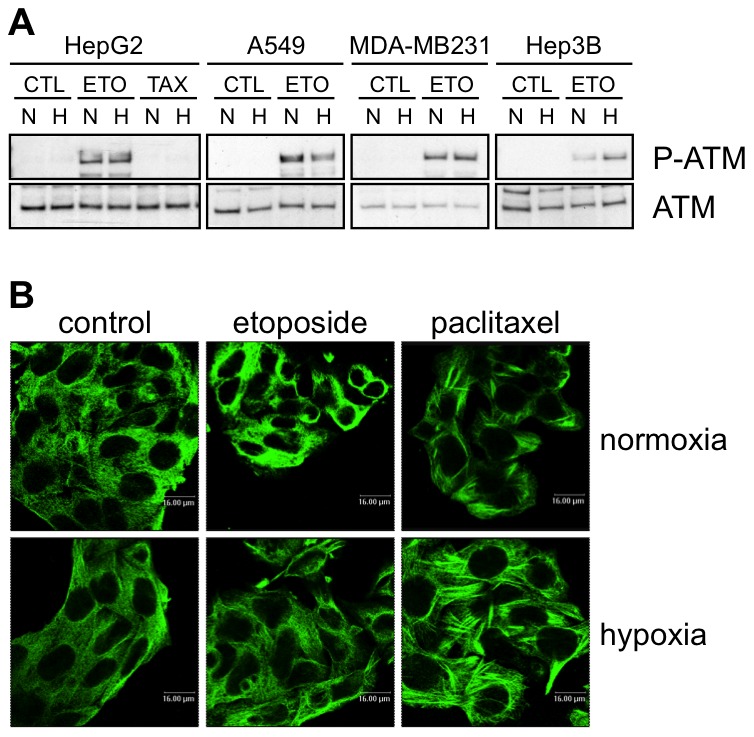
Effects of hypoxia on drug-induced damage. (A) Effect of hypoxia, etoposide and paclitaxel on the abundance and phosphorylation of ATM. HepG2, A549, MDA-MB231 and Hep3B cells were incubated 1 hour under normoxia (N, 21% O_2_) or hypoxia (H, 1% O_2_) in the presence or not (CTL) of etoposide (ETO, 100 µM in Hep3B cells and 50 µM in the other cell types) or paclitaxel (TAX, 10 µM) in HepG2 cells. ATM and P-ATM were detected in nuclear protein extracts by western blotting using specific antibodies. One experiment representative out of three. Uncropped western blots are presented in Figure S1. (B) Effect of hypoxia, etoposide and paclitaxel on microtubules. HepG2 cells were incubated 16 hours under normoxia (21% O_2_) or hypoxia (1% O_2_) in the presence or not of etoposide (50 µM) or paclitaxel (10 µM). After the incubation, cells were fixed, permeabilised and stained for alpha-tubulin using a specific antibody. Observation was performed using a confocal microscope with a constant photomultiplier.

Hypoxia is frequently described to inhibit the damaging effect of chemotherapeutic agents [15]. In order to check if hypoxia inhibits etoposide-induced DNA damage and/or paclitaxel-induced microtubule stabilisation or if it acts at a level downstream of the damage caused by the drug, ATM (ataxia telangiectasia mutated) phosphorylation was studied 1 hour after etoposide incubation and the morphology of the microtubule fibres was studied after 16 hours paclitaxel exposure. Etoposide triggers cell death by inducing double strand DNA breaks that lead to ATM activation. The results showed that it indeed induced the phosphorylation of ATM in the four cell types. No effect of hypoxia was observed on the induction of P-ATM, even in HepG2 and MDA-MB231 cells in which hypoxia decreased the etoposide-induced apoptosis (Figure 2A). In HepG2 cells that are protected by hypoxia against paclitaxel-mediated cell death, hypoxia did not seem to prevent the formation of thick microtubule fibres induced by paclitaxel (Figure 2B and data not shown). Altogether, these results showed that hypoxia protects cells downstream of the drug-induced damage.

**Figure 3:**
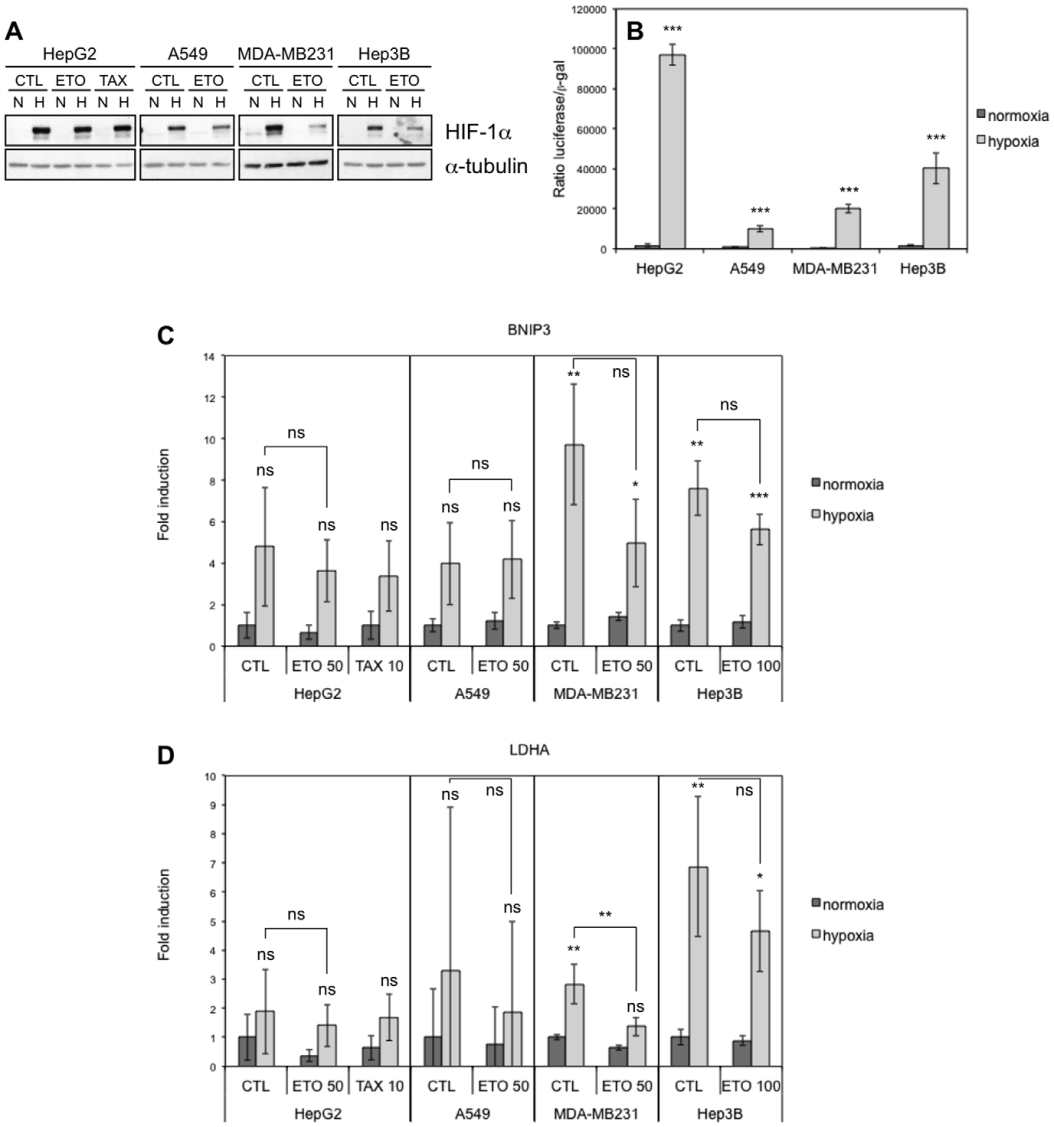
Effect of hypoxia, etoposide and paclitaxel on HIF-1alpha abundance and HIF-1 activity. HepG2, A549, MDA-MB231 and Hep3B cells were incubated 16 hours under normoxia (N, 21% O_2_) or hypoxia (H, 1% O_2_) (A-D) in the presence or not (CTL) of etoposide (ETO, 100 µM in Hep3B cells and 50 µM in the other cell types) or paclitaxel (TAX, 10 µM) in HepG2 cells (A, C, D). (A) HIF-1alpha was detected in total cell extracts by western blotting using a specific antibody. Alpha-tubulin was used as loading control. One experiment representative out of three. Uncropped western blots are presented in Figure S1. (B) Before incubation, cells were co-transfected with the pGL3-(PGK-HRE6)-tk-luc reporter plasmid encoding the firefly luciferase and the pCMVß normalisation plasmid. Results are expressed as mean of the ratio between firefly luciferase activity and ß-galactosidase activity ±1 SD (n = 3). Statistical analysis was carried out with ANOVA 1. ***: P<0.001. Symbols are a comparison between the normoxic and hypoxic conditions of the same cell type. (C, D) After incubation, total RNA was extracted, submitted to reverse transcription and then to amplification in the presence of SYBR Green and specific primers for BNIP3 (**c**) or LDHA (D). RPL13A was used as housekeeping gene for data normalization. Data are given in fold-induction as the mean ±1 SD (n  = 3). Statistical analysis was carried out with ANOVA 1. ns: non-significant; *: P<0.05; **: P<0.01; ***: P<0.001. Symbols above the hypoxic columns are a comparison with the corresponding normoxic condition (with the same drug).

Many protective effects of hypoxia are mediated by the HIF-1 (hypoxia-inducible factor-1) transcription factor, which is a heterodimer composed of one constitutively expressed subunit, HIF-1beta, and one subunit stabilised only under hypoxic conditions, HIF-1alpha [5]. We therefore investigated if this factor was effectively activated by hypoxia in the cell lines in which hypoxia does not have a protective effect (Figure 3). Hypoxia induced the accumulation of HIF-1alpha but etoposide decreased this accumulation in A549, MDA-MB-231 and Hep3B cells. In HepG2 cells, etoposide had no or a slight inhibiting effect according to the experiment. The effect of etoposide on HIF-1alpha protein level has already been observed [16]. As evaluated by luciferase reporter, HIF-1 was active in each cell type during hypoxia and this correlates with the mRNA abundance of two of its target genes (LDHA and BNIP3).

In conclusion, the fact that hypoxia protects HepG2 and MDA-MB231 cells against death but not A549 and Hep3B cells could not be explained by a difference in etoposide- or paclitaxel-induced damage or in HIF-1 activation.

### Expression Profile of mRNAs Involved in Apoptosis

**Figure 4:**
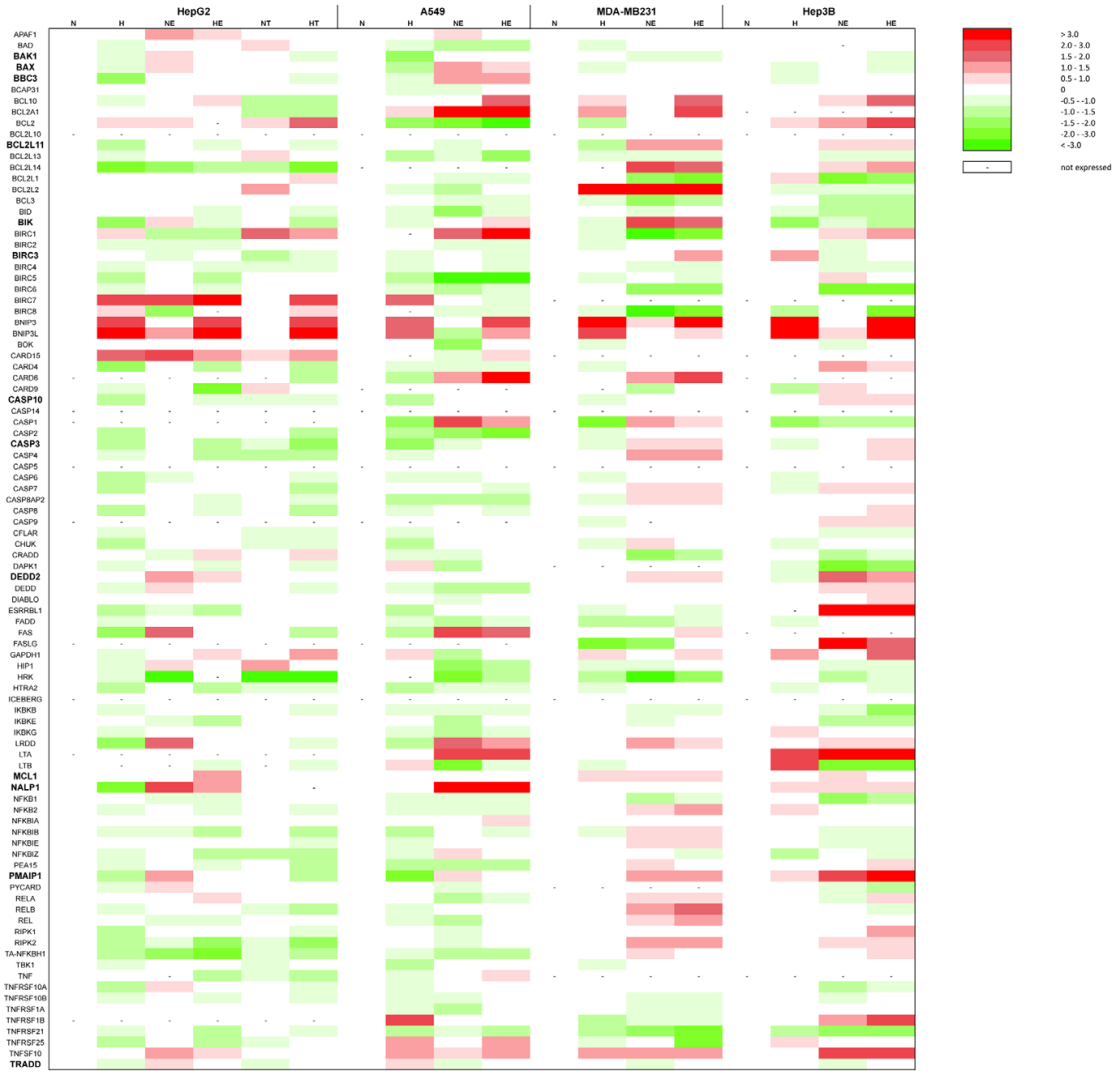
Effect of hypoxia, etoposide and paclitaxel on the mRNA expression level of genes involved in the apoptotic pathway. Results were obtained using “TLDA Human Apoptosis Panel” (Applied Biosystems) (TLDA). HepG2, A549, MDA-MB231 and Hep3B cells were incubated 16 hours under normoxia (N, 21% O_2_) or hypoxia (H, 1% O_2_) in the presence or not of etoposide (E, 100 µM in Hep3B cells and 50 µM in the other cell types) or paclitaxel (T, 10 µM) in HepG2 cells. After incubation, total RNA was extracted, submitted to reverse transcription and then to TLDA analysis. 18S was used as housekeeping gene for data normalization. Actual numerical values are provided in Table S2. Genes shown in bold are genes whose expression was validated by qRT-PCT.

Results from Figure 2 suggest that the differential effect of hypoxia on cell death occurs downstream of the induction of cell damage. We thus hypothesised that hypoxia influences the signal transduction pathways that trigger apoptosis. These pathways are notably depending on p53 but also on other transcription factors. Gene expression studies were thus performed to find genes whose expression is differentially regulated by hypoxia in protected cells in comparison to non-protected cells. Taqman Low Density Arrays (Applied Biosystems) enabling the study of the abundance of 93 mRNAs known to be involved in the apoptosis process (as well as 3 housekeeping mRNAs) were used. The results are presented as a heatmap in Figure 4 while the numerical values are provided in Table S2. As expected, we observed the induction of HIF-1 target genes (BNIP3, BNIP3L and GAPDH) after 16 hours hypoxia in the four cell types. Induction of p53 target genes (APAF-1, BAK1 and BAX) was observed in HepG2 and A549 cells after 16 hours etoposide incubation but not in the p53 mutated (MDA-MB231 cells) or null (Hep3B cells) cells. The p53 target gene PMAIP1 (NOXA) was induced by etoposide in all cell types.

**Figure 5:**
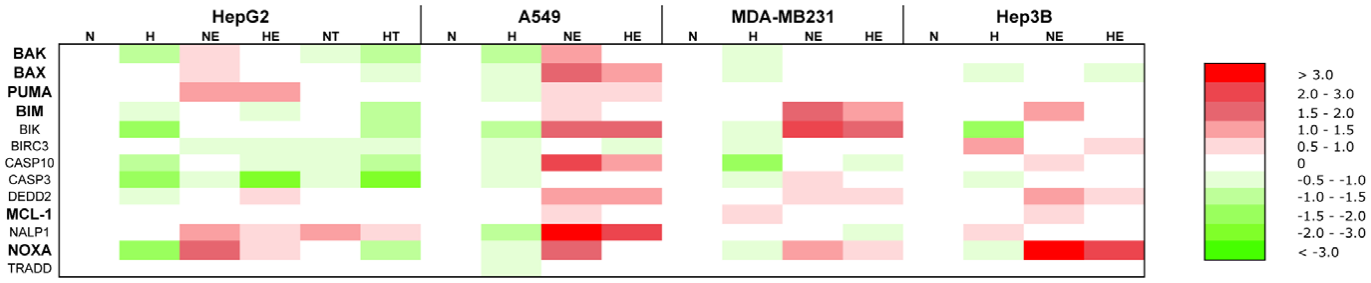
Effect of hypoxia, etoposide and paclitaxel on the mRNA expression level of genes involved in the apoptotic pathway. HepG2, A549, MDA-MB231 and Hep3B cells were incubated 16 hours under normoxia (N, 21% O_2_) or hypoxia (H, 1% O_2_) in the presence or not of etoposide (E, 100 µM in Hep3B cells and 50 µM in the other cell types) or paclitaxel (T, 10 µM) in HepG2 cells. After incubation, total RNA was extracted, submitted to reverse transcription and then to real-time PCR in the presence of SYBR Green and specific primers. Actual numerical values are provided in Table S3. Genes shown in bold are genes whose expression was assessed at the protein level by western blot analyses.

We then sought for genes whose expression profile is parallel to the cell death profile in each of the four cell types, i.e. genes whose expression was downregulated by hypoxia in HepG2 and MDA-MB231 cells and unaffected or increased in A549 and Hep3B cells, or the vice-versa. However, no such expression profile could be observed. Interestingly, the expression of BCL2L11 (also called BIM), BIK, CASP10, CASP3, DEDD2, NALP1 and TRADD was decreased by hypoxia in etoposide-incubated HepG2 cells while their expression was increased or unmodified in A549 cells, correlating with the apoptotic profile of these two cell types. Moreover, BIRC3 and MCL1 expression profile was inverse of the apoptotic profile for HepG2 and A549 cells. The expression profile of these genes was validated by single real-time RT-PCR reactions. The results are presented as a heatmap in Figure 5 while the numerical values are provided in Table S3. The profile of BAK1, BAX, BBC3 (also called PUMA) and PMAIP1 (also called NOXA) was also validated since they are well-known mediators of the etoposide-induced cell death. Most of the results obtained using Taqman Low Density Arrays were confirmed. It must however be noted that the expression of nearly all studied genes was decreased by hypoxia in all the etoposide-exposed cells.

### Expression Profile of Proteins Involved in Apoptosis

**Figure 6:**
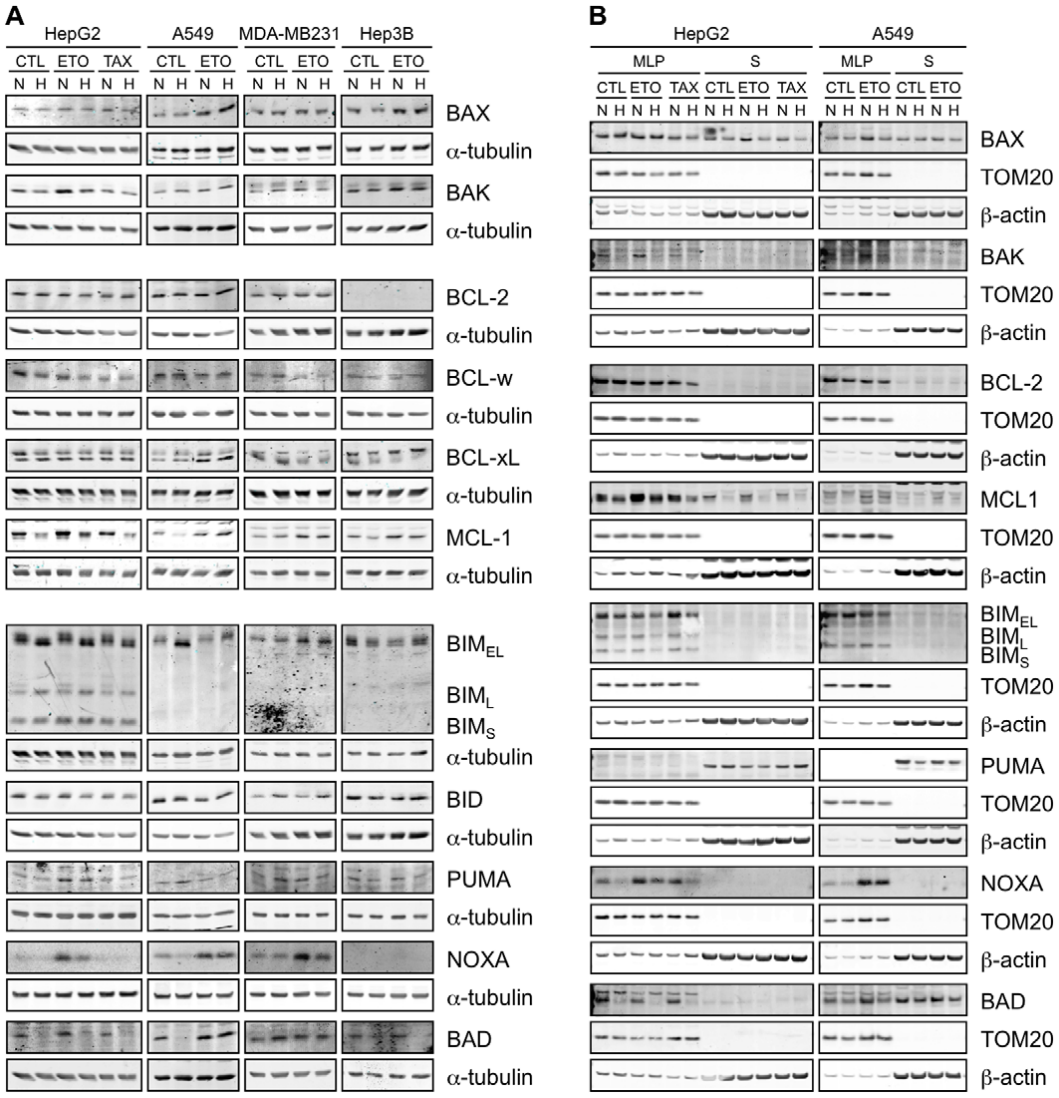
Effect of hypoxia, etoposide and paclitaxel on the abundance and localisation of BCL-2 family proteins. HepG2, A549, MDA-MB231 and Hep3B cells were incubated 16 hours under normoxia (N, 21% O_2_) or hypoxia (H, 1% O_2_) in the presence or not (CTL) of etoposide (ETO, 100 µM in Hep3B cells and 50 µM in the other cell types) or paclitaxel (TAX, 10 µM) in HepG2 cells. (A) Proteins were detected in total cell extracts by western blotting, using specific antibodies. alpha-tubulin was used as loading control. One experiment representative out of three. Uncropped western blots are presented in Figure S1. (B) After the incubation, subcellular fractionation was performed and proteins were detected in the MLP (mitochondria-lysosome-peroxisome) and S (cytosolic) fractions by western blotting, using specific antibodies. TOM20 and β-actin were used as loading controls for the MLP and S fractions respectively. One experiment representative out of three. Uncropped western blots are presented in Figure S1.

In addition to be regulated by changes in gene expression, most of the proteins involved in apoptosis are known to be also regulated at the post-translational level [2]. Etoposide and paclitaxel induce apoptosis by activating mainly the intrinsic pathway [17], [18], [19], which is regulated by the abundance and the subcellular localisation of proteins of the BCL-2 family. We therefore studied the protein abundance of BCL-2 family proteins in the four cell types as well as the subcellular localization of some of them in HepG2 and A549 cells (Figure 6).

We first studied the pro-apoptotic BAX and BAK proteins. BAX total protein level was slightly increased by etoposide in HepG2, A549 and Hep3B cells but remained unaffected in MDA-MB231 cells. Hypoxia slightly decreased BAX abundance in HepG2 cells but had no effect in other cell types. This protein was present in the MLP (mitochondria-lysosome-peroxisome) fraction as well as in the cytosol of HepG2 and A549 cells. Etoposide increased BAX abundance in the cytosol of HepG2 cells and in the MLP fraction of A549 cells; this increase was prevented by hypoxia. BAK total protein abundance was increased by etoposide in HepG2, MDA-MB231 and Hep3B cells and this increase was partly prevented by hypoxia in the three cell lines. In A549 cells, no effect of hypoxia or etoposide was observed. BAK is only present in the MLP fraction and its expression was increased by etoposide in HepG2 and A549 cells and strongly decreased by hypoxia. In conclusion, in HepG2 and MDA-MB231 cells in which hypoxia protects against cell death, we observed that hypoxia decreased the protein abundance of the BAK pro-apoptotic protein. However, a decrease in BAK protein level was also observed in Hep3B cells, which are not protected by hypoxia.

We then studied the abundance of anti-apoptotic proteins of the BCL-2 family: BCL-2, BCL-w, BCL-xL and MCL-1. BCL-xL protein level was increased by etoposide in A549 cells but, apart of that, the BCL-2 and BCL-xL protein levels were not affected by etoposide or hypoxia in the four cell lines. BCL-2 seemed not to be expressed in Hep3B cells and was only detected in the MLP fraction of HepG2 and A549 cells. Hypoxia decreased BCL-w protein level in HepG2 cells but not in the other cell lines. Finally the MCL-1 abundance was increased by etoposide in the four cell lines (in both fractions in HepG2 and A549 cells) and decreased by hypoxia in HepG2 and Hep3B cells. The results obtained by subcellular fractionation correlated with the results obtained with total extracts. In conclusion, in HepG2 cells in which hypoxia protects against cell death we observed that, on the contrary to what we could have expected, hypoxia decreased the protein abundance of anti-apoptotic proteins such as BCL-w or MCL-1. We also surprisingly observed that etoposide induced an increase in the anti-apoptotic protein MCL-1 level in the four cell lines.

Finally, we followed the abundance of the “BH3-only” proteins BIM (3 isoforms), BID, PUMA, NOXA and BAD. In Hep3B cells, NOXA protein was not detected and BAD protein was detected at a very low level in comparison to the other cell types. Etoposide increased BIM_EL_ abundance in MDA-MB231 cells but, apart of this, etoposide or hypoxia did not have marked effects on BIM total protein levels. An increase in BIM_EL_ abundance is sometimes observed after incubation of A549 cells under hypoxia but this observation was not always reproducible. BIM was only detected in the MLP fraction and its abundance was decreased by hypoxia for the 3 isoforms in HepG2 cells, by etoposide in A549 cells for the BIM_EL_ isoform and increased by paclitaxel in HepG2 cells for the BIM_EL_ isoform. In total extracts of HepG2 and A549 cells, changes in electrophoretic migration in Tris-glycine SDS-PAGE gels were detected. Indeed, when combined to etoposide, hypoxia increased BIM_EL_ migration while it was not the case in the 3 other cell types. We hypothesised that hypoxia-induced changes in post-translational modifications of BIM_EL_ in HepG2 cells. Since ERK (extracellular regulated kinase) and JNK (c-Jun N-terminal kinase) are known to phosphorylate BIM [20], [21], the effect of etoposide and/or hypoxia on the phosphorylation of these two kinases was investigated. The results showed that ERK phosphorylation was enhanced by etoposide in A549 cells while JNK phosphorylation was enhanced in HepG2 and in A549 cells. This phosphorylation was inhibited by hypoxia in etoposide-stimulated HepG2 cells, while it was only slightly inhibited in A549 cells (Figure S2). No effect of etoposide and/or hypoxia could be detected in MDA-MB231 or Hep3B cells.

BID abundance was slightly decreased by hypoxia in HepG2 cells but increased by hypoxia in MDA-MB-231 cells and unaffected in the two other cell lines. tBID (truncated BID) was not detected. PUMA abundance was increased by etoposide in HepG2 cells alone and slightly decreased by hypoxia in HepG2 and Hep3B cells. PUMA was only detected in the cytosolic fraction and its abundance was unaffected by etoposide or hypoxia in HepG2 cells and a little bit decreased by hypoxia in A549 cells. Etoposide strongly increased NOXA protein level in HepG2, A549 and MDA-MB231 cells and this was partly prevented by hypoxia in HepG2 and MDA-MB231 cells only. This protein abundance profile is very interesting since it correlates with the apoptotic profile. These observations were confirmed by the results obtained by subcellular fractionation with NOXA being only detected in the MLP fraction. We also observed that, in this fraction, paclitaxel increased NOXA protein level. Etoposide slightly increased BAD protein abundance in the four cell types and hypoxia prevented this effect in HepG2 and Hep3B cell types only. In HepG2 cells, BAD was mainly observed in the MLP fraction with a strong decrease in its abundance under hypoxia. In A549 cells, BAD was observed in both fractions and a slight decrease in its abundance was also detected under hypoxia.

### Role of BIM, NOXA and BAD in the Etoposide-induced Apoptosis of HepG2 Cells

We observed that the abundance of several BH3-only proteins was decreased by hypoxia in cells that are protected by hypoxia against cell death. We thus studied the role of three of them in the regulation of apoptosis for the following reasons: NOXA, since its abundance varied in parallel to the apoptotic profile in the four cell types; BIM, since it displayed a different electrophoretic mobility under hypoxia vs normoxia in etoposide-exposed HepG2 cells; and BAD since its abundance was strongly decreased by hypoxia in HepG2 cells. Specific siRNAs were used in HepG2 cells incubated in the presence of etoposide under normoxia or hypoxia.

**Figure 7:**
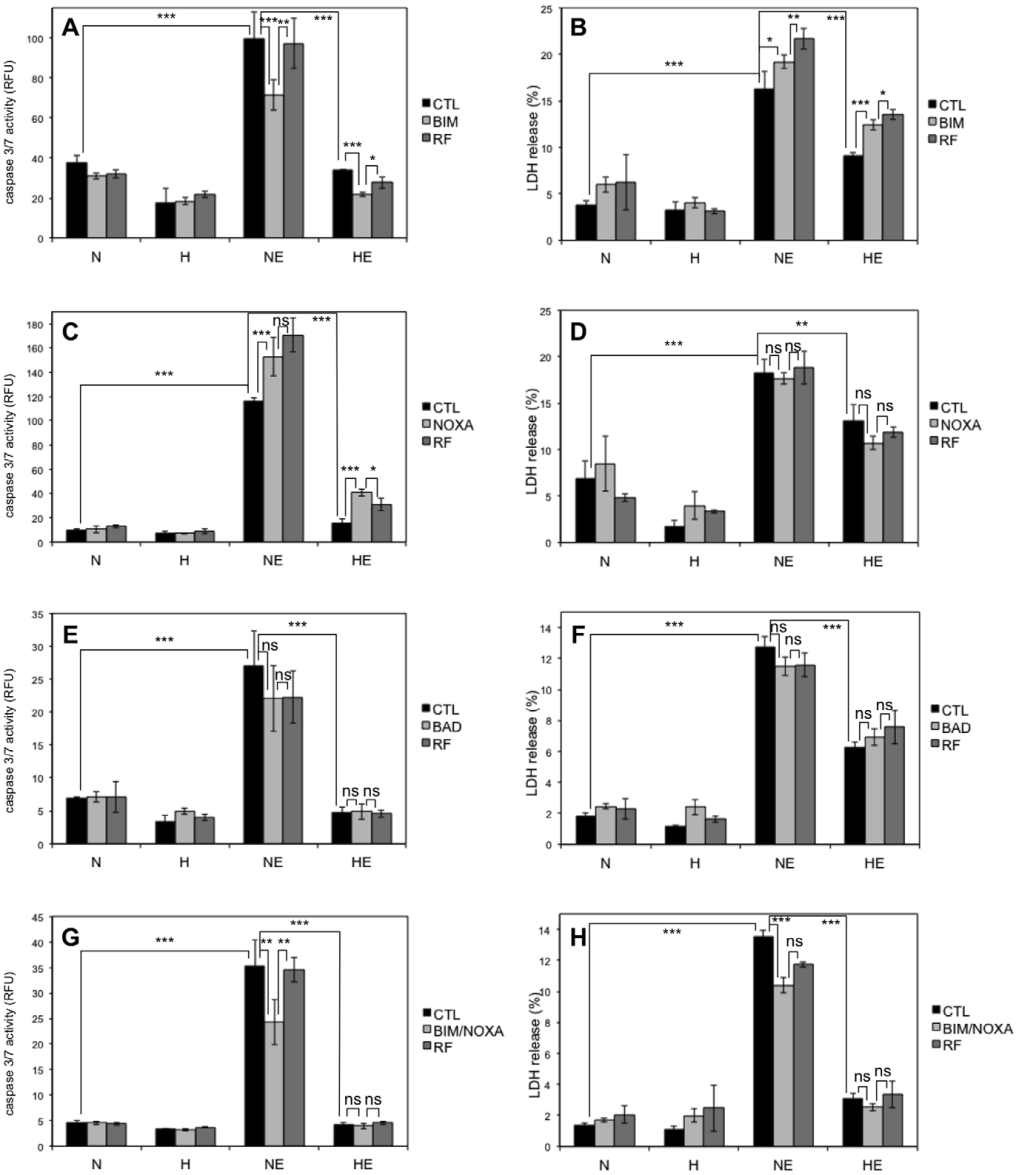
Effect of BH3-only proteins silencing on the etoposide-induced cell death . HepG2 cells were transfected with 50 nM BIM (A, B), NOXA (C, D) or BAD (E, F) siRNAs, or 25 nM BIM combined with 25 nM NOXA (G, H), or 50 nM RISC-free (RF) control siRNA or left untransfected (CTL) for 24 hours. 6 hours later (or 30 hours later for A and B), cells were incubated under normoxia (N, 21% O_2_) or hypoxia (H, 1% O_2_) with (ETO) or without (CTL) etoposide (50 µM) for 16 (A, C, E, G) or 40 hours (B, D, F, H). (A, C, E, G) Caspase-3/7 activity was assayed by measuring the fluorescence of free AFC released from the cleavage of the caspase-3/7 specific substrate Ac-DEVD-AFC. Results are expressed in relative fluorescence units (RFU) as means ±1 SD (n  = 3). (B, D, F, H) LDH release was assessed. Results are presented in percentages as means ±1 SD (n  = 4, but n  = 3 in B for the condition HE CTL and in **f** for the condition H BAD). (A-H) Statistical analysis was carried out with ANOVA 1. ns: non-significant; *: P<0.05; **: P<0.01; ***: P<0.001.

The efficiency of BIM, NOXA and BAD knockdown by siRNA is shown in Figure S3. The effects of the knockdown of each of these proteins were investigated on caspase-3/7 activity after 16 hours incubation and on LDH release after 40 hours incubation (Figure 7). No effect of NOXA or BAD knockdown was observed on apoptosis neither on overall cell death. However, BIM knockdown induced a decrease in caspase-3/7 activity in cell incubated with etoposide under normoxia as compared with untransfected cells or with cells transfected with a negative control. However, this decrease in caspase activity was not translated into a decrease in overall cell death as measured by LDH release assay. BIM is therefore probably partly involved in etoposide-induced cell death but not alone. We therefore investigated the effect of the combined silencing of BIM and NOXA proteins. BIM and NOXA combined knockdown induced a significant decrease in caspase-3/7 activity as well as in LDH release in cells incubated with etoposide under normoxia as compared with untransfected cells. We thus concluded that BIM and NOXA are important mediators of etoposide-induced cell death and that the difference in post-translational modification of BIM and the decrease in NOXA protein level observed in hypoxic HepG2 cells at least in part contribute to the decreased cell death observed under hypoxia.

**Figure 8:**
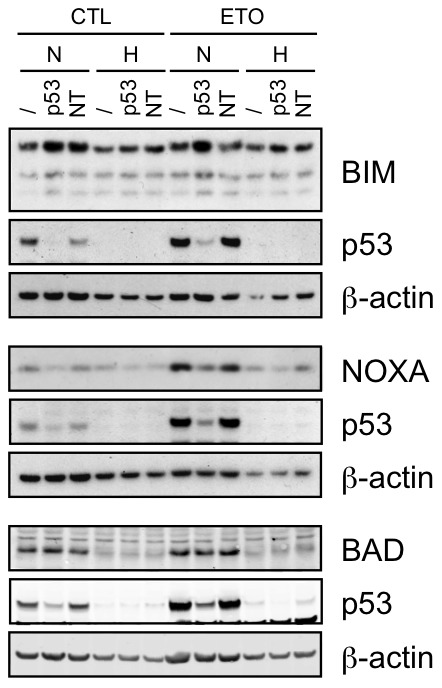
Effect of p53 silencing on the expression of BIM, NOXA and BAD. HepG2 cells were transfected with 50 nM p53 siRNA (p53) or non-targeting control siRNA (NT) or left untransfected (/) for 24 hours. Minimum 6 hours later, cells were incubated under normoxia (N, 21% O_2_) or hypoxia (H, 1% O_2_) with (ETO) or without (CTL) etoposide (50 µM) for 16 hours. Proteins were detected in total cell extracts by western blotting, using specific antibodies. ß-actin was used as loading control. One experiment representative out of three. Uncropped western blots are presented in Figure S1.

Since we already evidenced a potential role for p53 in the cell behaviour observed under hypoxia in HepG2 cells [22], we checked if BIM, NOXA and BAD expression were dependent on p53 by using siRNAs directed against p53 (Figure 8). As expected, p53 knockdown strongly decreased NOXA abundance, which is known to be a p53 target gene, but did not affect BIM and BAD abundance. Hypoxia therefore probably induced p53-dependent and -independent pathways to modulate BH3-only protein abundance and post-translational modifications.

## Discussion

Hypoxic areas are frequently observed in solid tumours [23]. Due to uncontrolled tumour growth, cells in the centre of the tumour are indeed often too far away from the blood vessels to receive enough oxygen. Disorganised tumour vasculature further impairs oxygen delivery to cancer cells. Severe hypoxia may induce cell death, leading to necrotic areas within tumours. On the contrary, mild hypoxia enhances tumour cell survival and induces resistance to chemotherapy [7]. There is no gold standard for measuring hypoxia. Eppendorf measurement of pO_2_ has been used in human tumours but this method is invasive. Actual values are available for head and neck, cervix and prostate carcinomas. They usually range in between 1 and 10 mm Hg (0.14 to 1.4% O_2_) (for a review see [24]). Actual values for other types of human cancers are not available. Recent studies have focused on molecular markers of hypoxia (HIF-1α or CAIX) that demonstrate the presence of hypoxic areas within the tumours and not within healthy tissues but these surrogate markers do not give absolute values. Similarly, pimonidazole staining also gives an indication of the presence of hypoxia but not absolute values. According to these data, most of the work published in the literature is performed at 1% O_2_ as the “standard” hypoxia. Moreover, biological responses to hypoxia are optimal at 1% O_2_. This is for example the case for the major regulator of these adaptive responses, the transcription factor HIF-1. At much lower pO_2_ level, other responses are triggered that usually lead to cell death (for reviews see [25], [26], [27]).

Cell responses to hypoxia obviously depend on the severity and the duration of the decreased oxygen availability but also on the cell type. For example, hypoxia (1% oxygen) protects HepG2 cells against etoposide-induced apoptosis as well as MDA-MB231 cells against paclitaxel-induced apoptosis but has no effect or even aggravates the apoptosis induced by etoposide in A549 and MCF-7 cells as well as the epirubicin-induced apoptosis of MDA-MB231 cells [8], [9], [10]. In order to better understand why hypoxia aggravates the chemotherapy-induced cell death in some cell types while it protects against it in other cell types, we studied the responses of seven cell types exposed to chemotherapy under hypoxia. Cells from different organs and with different p53 status were chosen. p53 mutation or inhibition is indeed a main cause of chemoresistance [28] and it has been observed that hypoxia can increase or decrease p53 abundance and/or activity [29]. p53 therefore probably plays an important role in the choice between life and death of hypoxic cancer cells. Furthermore, the effects of five different agents targeting different cell components to induce cell death were compared. We observed that hypoxia generally prevented drug-induced apoptosis, but hypoxia has sometimes no effect, and in rare cases it could increase drug-induced cell death. Interestingly, in most cases, the effect of hypoxia was the same for all the drugs in one cell type. We also observed that hypoxia can protect p53 wild-type cells as well as p53 mutated cells against cell death.

In order to identify genes or proteins affected by hypoxia and involved in the choice between life and death, four cell types responding differently towards apoptosis under hypoxia were selected: HepG2 (p53 wild-type cells that are protected by hypoxia), A549 (p53 wild-type cells that are not protected by hypoxia), MDA-MB231 (p53 mutated cells that are protected by hypoxia) and Hep3B cells (p53 null cells that are not protected by hypoxia). Death was induced by etoposide in the four cell lines. In addition, the effects of paclitaxel were compared to the ones of etoposide in HepG2 cells. We showed that hypoxia activated the HIF-1 transcription factor in all cell lines and that etoposide and paclitaxel were effective for inducing DNA and microtubule damage respectively during hypoxia. The effect of hypoxia on cell death therefore occurs downstream the initial damage and is not dependent of a different activation of HIF-1. The study of the abundance of 93 mRNAs known to be involved in the apoptosis process showed that most of these genes were downregulated by hypoxia in comparison to normoxia, in the presence of etoposide in the four cell types. We found no candidate mRNA whose variations of abundance in the different cells types could explain the cell death profile for the four cell types.

**Figure 9:**
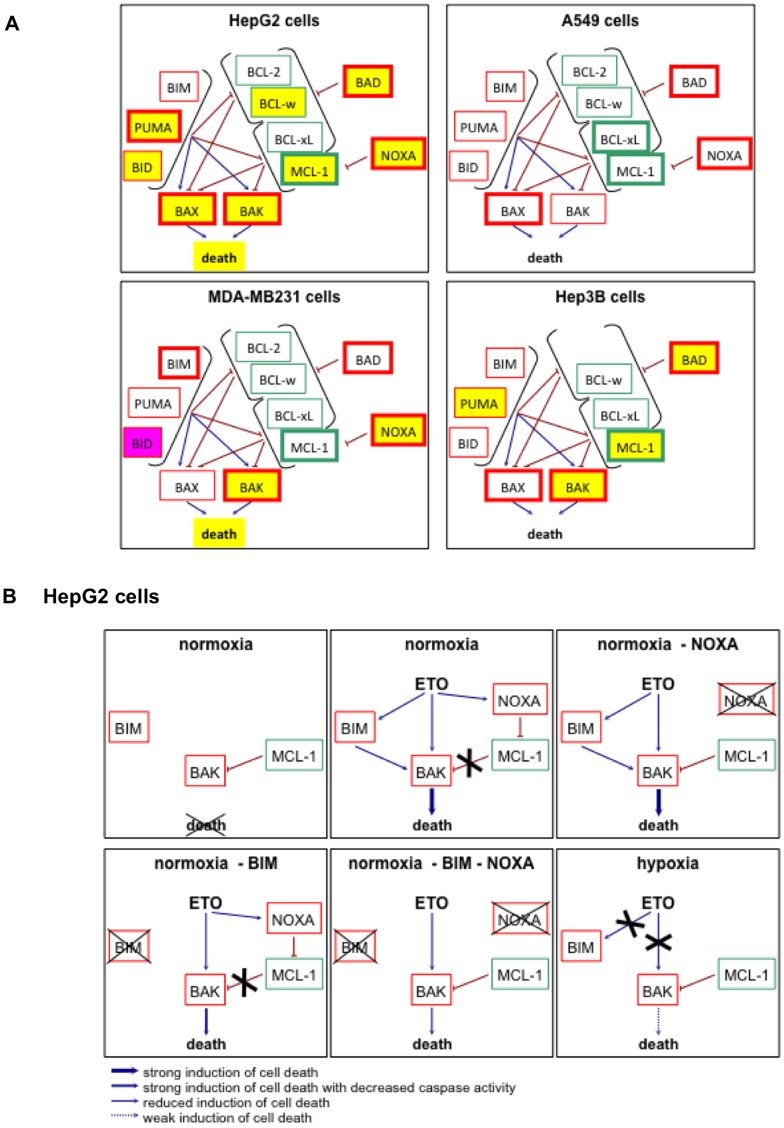
Schematic representation of the effects of hypoxia on the etoposide-induced effects on BCL2-family proteins. (A) Schematic representation of the results obtained in figure 4. Anti-apoptotic proteins are represented outlined in green and pro-apoptotic proteins are outlined in red. The activation arrows and inhibition signs come from the hypothesis of the interactions between BCL-2 family proteins as explained in [69], [70]. The proteins whose abundance is increased by etoposide are outlined with a thicker line. A yellow filling represents that, in the presence of etoposide, hypoxia decreased the abundance of the protein (or the death) as compared to normoxia. A pink filling represents that, in the presence of etoposide, hypoxia increased the abundance of the protein as compared to normoxia. (B) Schematic representation of the hypothetic relations between BIM, BAK, MCL-1 and NOXA. These possible mechanisms are represented for normoxic (21% O_2_) or hypoxic (1% O_2_) HepG2 cells incubated with or without etoposide (ETO; 50 µM) and with or without silencing (−) of NOXA and/or BIM. The hypothetic relations between these proteins are explained in details in the text. Anti-apoptotic proteins are represented outlined in green and pro-apoptotic proteins are outlined in red.

Apoptosis is strongly regulated by the abundance and localisation of proteins of the BCL-2 family [1]. Hypoxia is known to modulate the abundance and/or post-translational modifications of most of these proteins. For example, hypoxia upregulates the expression of anti-apoptotic genes and proteins of the family such as the MCL-1 and BCL-xL genes in a manner dependent of HIF-1, as well as BCL-2 [30]–[38]. Some reports described a hypoxia-induced increase in BAX protein level while others observed a decrease in BAX and/or BAK1 protein level [22], [33], [37], [39], [40], [41]. Several BH3-only proteins are also affected by hypoxia. NOXA is a HIF-1 target gene and a mediator of hypoxic cell death [42]. BIM and BMF expression can be suppressed by hypoxia in a HIF-1-dependent manner [43]. BID and BAD have been described to be upregulated or downregulated by hypoxia depending on the study [39], [40], [44]. Finally, hypoxia reduces BAD phosphorylation on Ser112 but increases its phosphorylation on Ser136 and Ser155 [45], [46]. Since hypoxia has frequently been described to affect BCL-2 family protein abundance, their protein expression level was assessed by western blot. The results are schematically represented in Figure 9A. As expected, etoposide increased the abundance of various pro-apoptotic proteins in the four cell types studied. It is for example the case of BAX and/or BAK, or BAD and NOXA. However, etoposide also induced the expression of the anti-apoptotic protein MCL-1. Our results show how much cell types can differently react to similar hypoxic conditions. In HepG2 cells that are markedly protected against cell death by hypoxia, hypoxia decreased the abundance of nearly all the pro-apoptotic BCL-2 family proteins while none of them are decreased in A549 cells that are not protected by hypoxia. We could therefore hypothesize that it is this differential effect of hypoxia on BCL-2 family protein abundance that is responsible for the different behaviour of these two cell types about apoptosis during hypoxia. For MDA-MB231 and Hep3B cells, such a hypothesis cannot be put forth. Indeed, in both cell types, hypoxia decreased the abundance of some pro-apoptotic proteins while their apoptotic response is different. The study of the abundance of BCL-2 family proteins is therefore not sufficient to understand the general apoptotic behaviour of a given cell type. It is however interesting to note that the NOXA upregulation by etoposide was partly prevented by hypoxia in cells that are protected against cell death (HepG2 and MDA-MB231 cells) while this was not the case in cells that are not protected by hypoxia (A549 and Hep3B cells, NOXA being not expressed in this latter cell type). The outcome of the network of interactions between proteins of the BCL-2 family evidently does not only depend on their abundance but also on their subcellular localisation, on their post-translational modifications and on binding partners. Moreover, BCL-2 family proteins are not the only regulators of apoptosis. Downstream regulation can also occur, such as by regulation of inhibitors of caspases for example.

With the aim to go further in the understanding of this complex network, we then focused on HepG2 cells that are strongly protected by hypoxia against etoposide-induced cell death. We chose to study the role of NOXA, BIM and BAD in the etoposide-induced apoptosis. These three proteins are indeed downregulated or modified under hypoxia. Etoposide induces DNA damage and is therefore known to induce cell death mainly via the p53 pathway. p53-induced apoptosis is principally triggered by PUMA and NOXA overexpression [47], [48]. Here we showed that both proteins are indeed upregulated by etoposide in HepG2 cells. However, unexpectedly, PUMA was not localised in the mitochondria-containing fraction. It is therefore possible that it does not initiate cell death. Etoposide also induced cell death and NOXA upregulation in p53 mutated or null cells. NOXA upregulation can be induced by p53-independent pathway such as via p73 [49], [50]. However, in etoposide-incubated HepG2 cells, NOXA knockdown did not decrease cell death. Besides the well-known role of NOXA, BIM has also been described to play a role in DNA damage-induced apoptosis [51]. The difference in electrophoretic mobility of BIM_EL_ between normoxia and hypoxia in the presence of etoposide probably reveals a change in post-translational modification that could correspond to a loss of phosphorylation during hypoxia. Isoelectric focusing two dimensional (IEF-2D) gel analysis of endogenously expressed BIM_EL_ showed at least 5 distinct spots in MCF-7 cells, which supposed that this protein is indeed subject to various post-translational modifications [52]. The main kinases known to phosphorylate BIM_EL_ are ERK1/2 and JNK. Following activation of the ERK1/2 pathway, BIM_EL_ could be phosphorylated on at least 3 residues and this would induce its dissociation from the BCL-2 family members, its ubiquitination and its degradation by the proteasome [20], [21]. The role of JNK-mediated phosphorylation of BIM_EL_ is still controversial since it can induce its degradation by the proteasome while in most cases it would rather enhance its pro-apoptotic activity notably by inducing a decrease in its affinity for DLC1 (dynein light chain 1) - a protein that can sequester BIM_EL_ in basal conditions – allowing BIM_EL_ to play a role at the mitochondrial level, and an increased interaction with BCL-2 [20], [21]. However, ERK1/2 and JNK have some common phosphorylation sites and it seems that BIM_EL_ stability and activity depends on a complex phosphorylation code that is cell type dependent [53], [54]. Other kinases have also been described to target BIM_EL_. It is for example the case for AKT, RSK 1/2 (ribosomal protein S6 kinase), ERK5 or CDK1 (cyclin-dependent kinase 1), which all promote cell survival either by inducing its sequestration away from the mitochondria or either by inducing its degradation [55], [56], [57], [58]. It is also the case for PKA (protein kinase A) or p38-MAPK (mitogen-activated protein kinase), which induce its stabilisation or enhance its pro-apoptotic activity [53], [59]. BIM_EL_ seems to be phosphorylated since its electrophoretic mobility was slower for normoxic HepG2 cells than for hypoxic cells. We showed that BIM knockdown decreased caspase-3/7 activity in HepG2 cells stimulated with etoposide without affecting the overall cell death as measured by LDH release. We hypothesize that in etoposide-stimulated HepG2 cells, BIM_EL_ is phosphorylated and is active in promoting apoptosis. This phosphorylation would be absent in hypoxic HepG2 cells, either due to a decreased activation of the kinase(s) or to the activation of a phosphatase such as PP2A (protein phosphatase 2A) which is known to dephosphorylate Bim_EL_ [52]. Both hypotheses could be right since PP2A is sometimes activated by hypoxia [60], [61] and since we observed that JNK phosphorylation was markedly inhibited by hypoxia in etoposide-stimulated HepG2 cells, while it was only very slightly inhibited in A549 cells. Finally, a major role of BAD in DNA-damage induced cell death is usually not described and we observed no effect of its knockdown on etoposide-induced cell death. Though NOXA knockdown had no impact on etoposide-induced cell death and BIM knockdown only affected the caspase activity but not the overall cell death, the combined loss of BIM and NOXA induced a decrease in caspase-3/7 activity and also induced a decrease in LDH release in cells incubated with etoposide under normoxia. These observations on HepG2 cell death are similar to the ones obtained by Kirschnek et al. when studying the spontaneous death of neutrophils obtained from knockout mice. They indeed observed no effect of BIM or NOXA single knockout on neutrophil cell death while the combined knockdown of these two proteins strongly protected these cells against cell death [3]. The difference in post-translational modification of BIM and the decrease in NOXA protein level observed in hypoxic HepG2 cells therefore probably contribute to the decreased cell death observed under hypoxia.

Our results suggest that BAK, BIM, NOXA and MCL-1 are the main mitochondrial players of etoposide-induced cell death that are regulated by hypoxia. Indeed, we previously evidenced that BAK is an important component of etoposide-induced cell death in HepG2 cells [22] and we here showed the involvement of BIM and NOXA. It is known that BCL-xL and MCL-1 are the main inhibitors of BAK, that the inhibition by MCL-1 can be relieved by NOXA [62], and that BIM can act as a direct activator of BAK [63]. Therefore, we hypothesize (Figure 9B) that, in control cells, a part of the BAK pool is inhibited by MCL-1. When the cells are incubated in the presence of etoposide, NOXA and BAK are upregulated and BIM is activated. NOXA inhibits MCL-1 and BAK is activated by BIM to induce cell death. In etoposide-incubated cells, NOXA knockdown has no effect on cell death since the free BAK pool is sufficient to promote cell death when it is activated by BIM. However, the absence of BIM partly prevented caspase activation. In cells in which BIM is silenced, NOXA knockdown decreased cell death since a part of BAK pool is inhibited by MCL-1 and since the BAK free pool is not activated by BIM. Hypoxia inhibited the etoposide-induced induction of BAK and NOXA as well as changed BIM post-translational modifications. These effects inhibit cell death since there is a smaller amount of BAK, that is not activated by BIM and that is partly inhibited by MCL-1, although MCL-1 abundance is also decreased.

The p53 protein level profile corresponds to the apoptotic profile of HepG2 and A549 cells [8] and p53 is probably an important node in the decision between life and death for hypoxic cancer cells [29]. Here, we showed that, in etoposide-stimulated HepG2 cells, NOXA abundance was dependent of p53 but that this was not the case for BIM and BAD abundance. These results correlate with the fact that NOXA is a p53-target gene [64], that BIM is probably not [65], while BAD is regulated by p53 in some cell types but not all [66]. We conclude therefore that hypoxia induced p53-dependent and -independent pathways to modulate BH3-only protein abundance, post-translational modifications as well as cell death.

In addition to modulate apoptosis, hypoxia may also influence autophagy, also called programmed type II cell death. Autophagy is a tightly regulated process by which selected components of a cell are degraded. It primarily functions as a cell survival adaptive mechanism during stress conditions. However, prolonged unresolved damage can also lead to cell death through excessive autophagy. Accumulating data indicate a role for autophagy in chemotherapy-induced cancer cell death [67]. Autophagy most commonly dampens treatment efficacy as it has been shown *in vitro* for different cell types. Hypoxia also triggers autophagy that participates to chemoresistance, notably by inducing BNIP3 expression [68]. A third adaptive response possibly triggered by hypoxia is the unfolded protein response. However, its possible role in chemoresistance is not yet well defined [26].

We showed that hypoxia affects the cell death of various cell types in a very different manner, influencing or not the chemotherapeutic efficacy. Hypoxia also modulates most proteins of the BCL-2 family in a cell type-dependent manner. In HepG2 cells that are protected by hypoxia against etoposide-induced cell death, many proteins of this family are downregulated, such as NOXA, or have different post-translational modifications, such as BIM. The combined loss of BIM and NOXA activity during hypoxia could partly account for chemoresistance. Hypoxia-induced chemoresistance is still an important problem for the success of therapies. Our results emphasize the need for a better comprehension of the factors causing these different cell behaviours in order to overcome the hypoxia-induced chemoresistance and ameliorate cancer therapy.

## Supporting Information

Figure S1 **Uncropped western blots for** **Figures 2** **,** **3** **,** **6** **and** **8** **and for Figures S2 and S3.**
(PDF)

Figure S2 **Effect of hypoxia, etoposide and paclitaxel on JNK and ERK phosphorylation.** HepG2, A549, MDA-MB231 and Hep3B cells were incubated 16 hours under normoxia (N, 21% O_2_) or hypoxia (H, 1% O_2_) in the presence or not (CTL) of etoposide (ETO, 100 µM in Hep3B cells and 50 µM in the other cell types) or paclitaxel (TAX, 10 µM) in HepG2 cells. Proteins were detected in total cell extracts by western blotting, using specific antibodies. Uncropped western blots are presented in the supplementary figure 1.
(PDF)

Figure S3 **Effect of BH3-only proteins silencing on their mRNA and protein levels.** HepG2 cells were transfected with 50 nM BIM (**a**, **f**), NOXA (**b**, **g**) or BAD (**c**, **h**) siRNAs, or 25 nM BIM combined with 25 nM NOXA siRNAs (**d**, **e**, **i**), or 50 nM RISC-free (RF) control siRNA or left untransfected (/) for 24 hours. 6 hours later (or 30 hours later for **a** and **f**), cells were incubated under normoxia (N, 21% O_2_) or hypoxia (H, 1% O_2_) with (ETO, E) or without (CTL) etoposide (50 µM) for 16 hours. (**a**, **b**, **c**, **d**, **e**) After incubation, total RNA was extracted, submitted to reverse transcription and to amplification in the presence of SYBR Green and specific primers (RT-PCR). RPL13A was used as housekeeping gene for data normalization. Data are given in fold induction (n = 1). (**f**, **g**, **h**, **i**) Proteins were detected in total cell extracts by western blotting, using specific antibodies. alpha-tubulin was used as loading control. Uncropped western blots are presented in Figure S1.
(PDF)

Table S1 **Sequences of the primers used for RT-real-time PCR.**
(PDF)

Table S2 **Effect of hypoxia, etoposide and paclitaxel on the mRNA expression level of genes involved in the apoptotic pathway.** Results were obtained using “TLDA Human Apoptosis Panel” (Applied Biosystems) (TLDA). HepG2, A549, MDA-MB231 and Hep3B cells were incubated 16 hours under normoxia (N, 21% O_2_) or hypoxia (H, 1% O_2_) in the presence or not of etoposide (E, 100 µM in Hep3B cells and 50 µM in the other cell types) or paclitaxel (T, 10 µM) in HepG2 cells. After incubation, total RNA was extracted, submitted to reverse transcription and then to TLDA analysis. 18S was used as housekeeping gene for data normalization. Data are given in fold-induction. Grey cell notify that the Ct value was >35 and should therefore not be considered as quantitative. “-” non expressed or not possible to calculate a fold change because the mRNA is not expression in controle cells. Genes shown in bold are genes whose expression was validated by qRT-PCT.
(PDF)

Table S3 **Effect of hypoxia, etoposide and paclitaxel on the mRNA expression level of genes involved in the apoptotic pathway.** Comparison of results obtained using “TLDA Human Apoptosis Panel” (Applied Biosystems) (TLDA) and single real-time RT-PCR reactions (RT-PCR). HepG2, A549, MDA-MB231 and Hep3B cells were incubated 16 hours under normoxia (N, 21% O_2_) or hypoxia (H, 1% O_2_) in the presence or not of etoposide (E, 100 µM in Hep3B cells and 50 µM in the other cell types) or paclitaxel (T, 10 µM) in HepG2 cells. After incubation, total RNA was extracted, submitted to reverse transcription and then to TLDA analysis or to amplification in the presence of SYBR Green and specific primers (RT-PCR). 18S and RPL13A were used as house keeping genes for data normalization of TLDA and single real-time RT-PCR reactions respectively. Data are given in fold-induction as the mean ±1 SD (n  = 3) is provided for single real-time RT-PCR reactions. Refer to Table S2 for complete TLDA results. Grey cell notify that the Ct value was >35 and should therefore not be considered as quantitative. “-” non expressed or not possible to calculate a fold change because the mRNA is not expression in control cells. Genes shown in bold are genes whose expression was assessed at the protein level by western blot analyses.
(PDF)
