# Supplementary material for: Hypoxia-Induced Modulation of Apoptosis and BCL-2 Family Proteins in Different Cancer Cell Types
Source: PLoS One. 2012 Nov 5;7(11):e47519. doi: 10.1371/journal.pone.0047519 (PMC3489905; doi:10.1371/journal.pone.0047519)

Supplementary figure 1

Western blots from figure 2

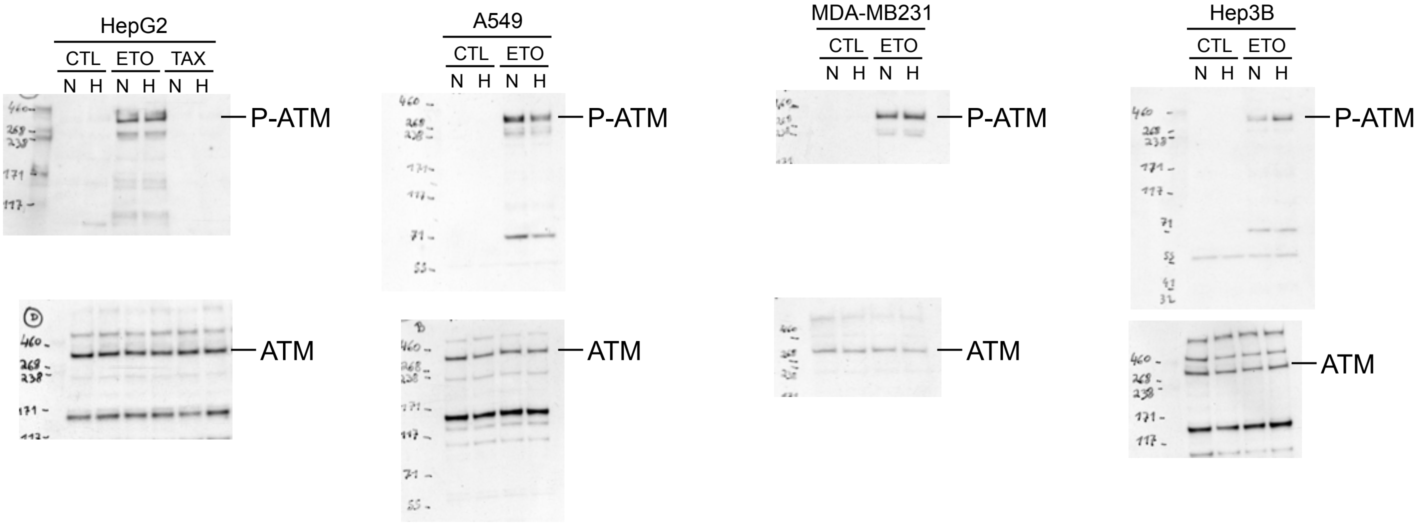

Western blots from figure 3

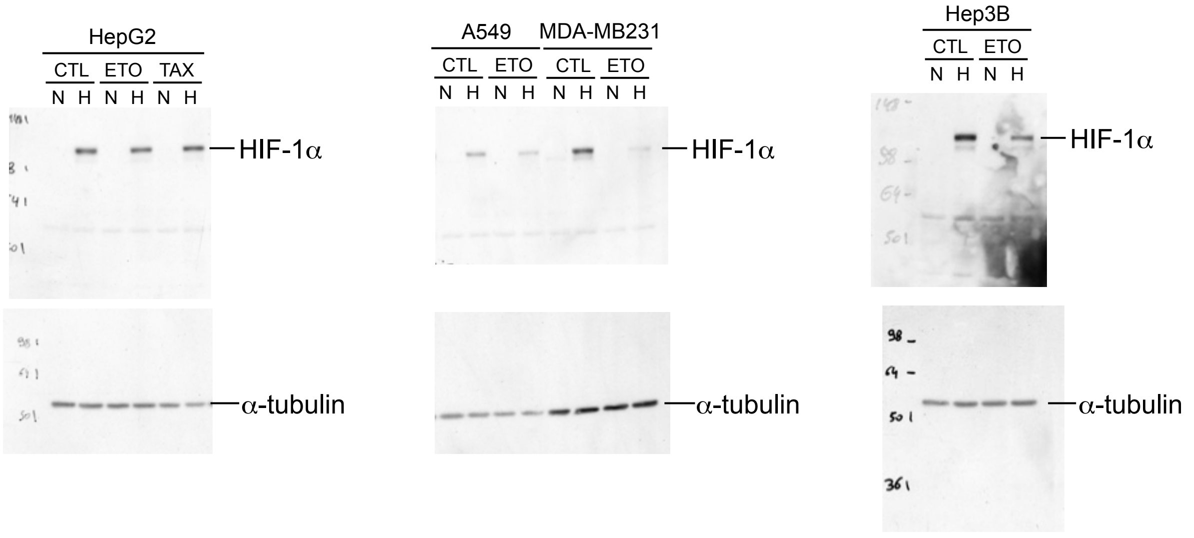

Western blots from figure 4

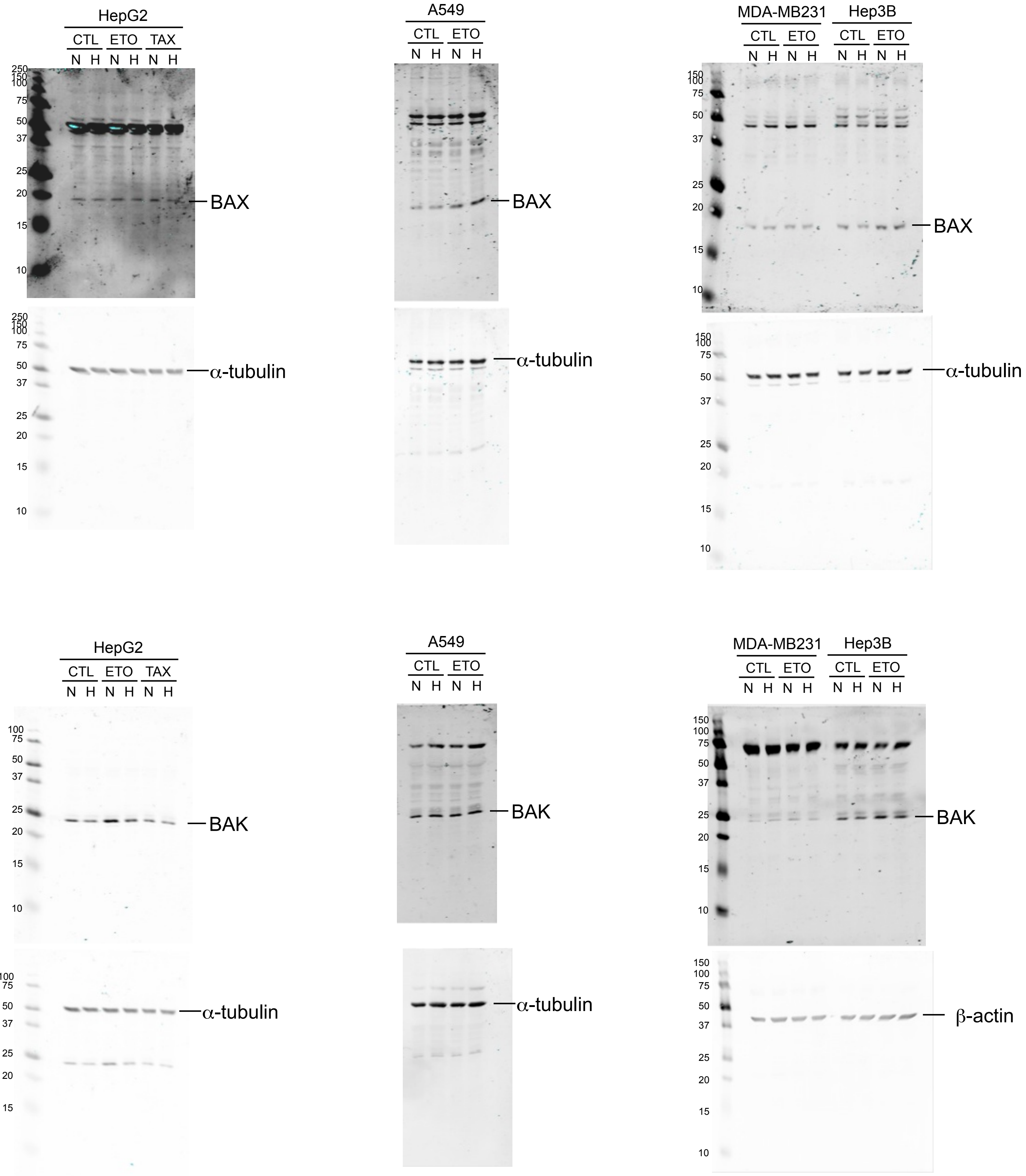

Western blots from figure 4

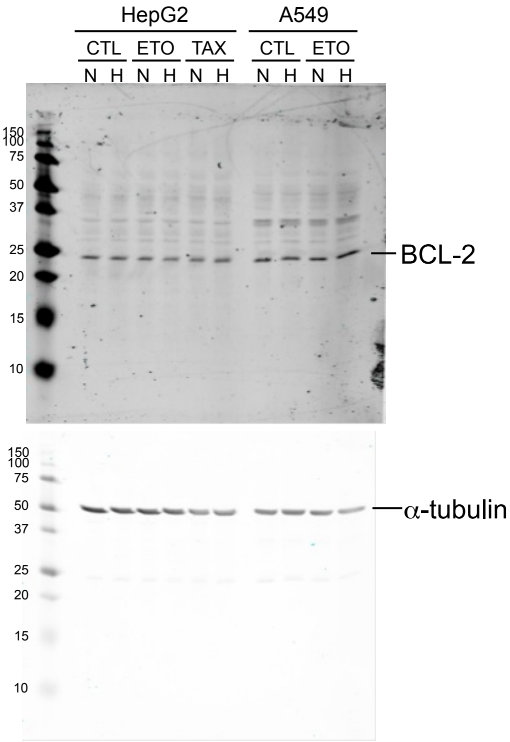

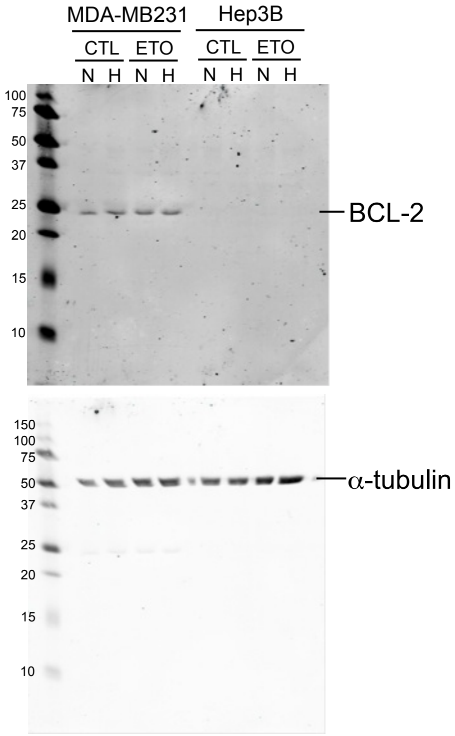

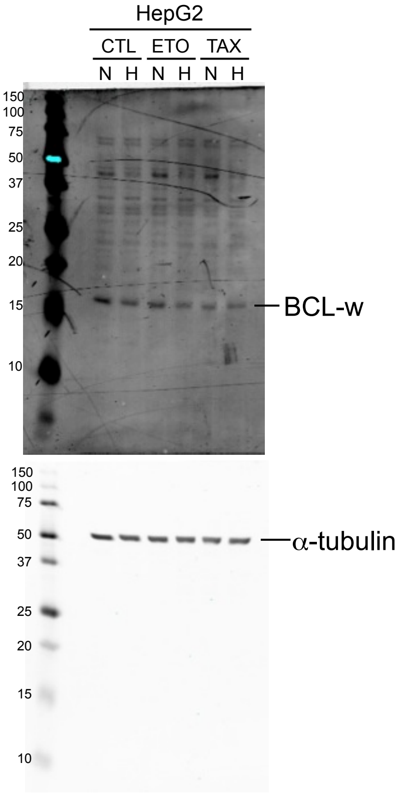

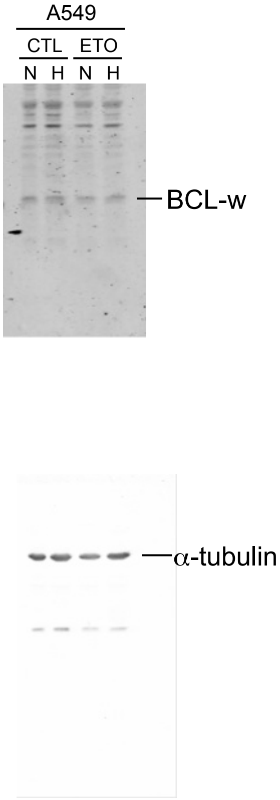

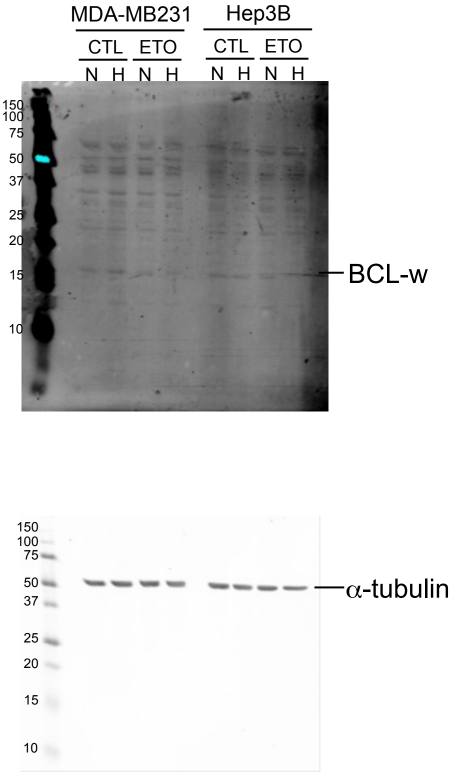

Western blots from figure 4

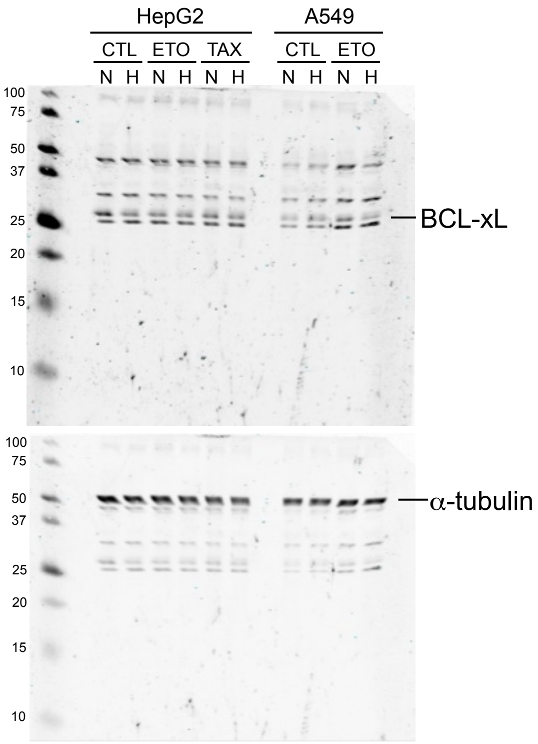

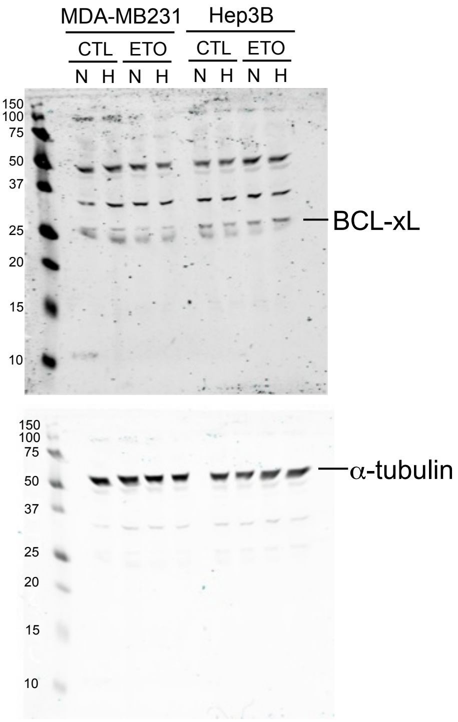

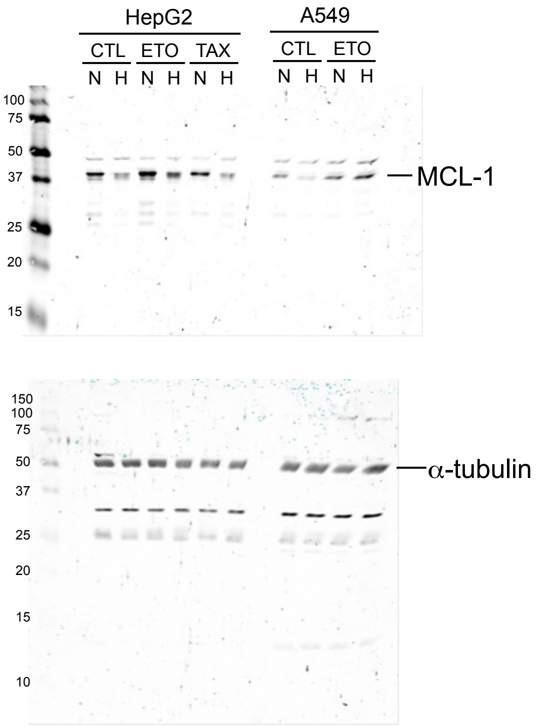

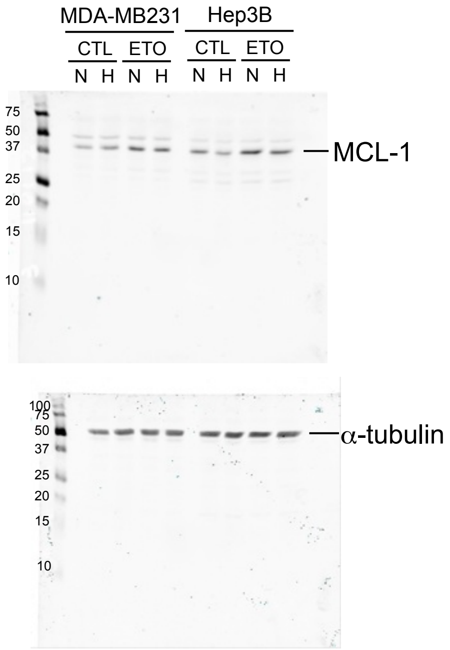

Western blots from figure 4

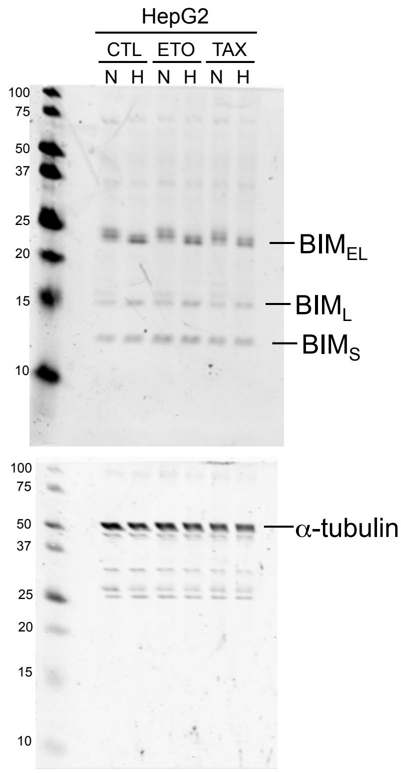

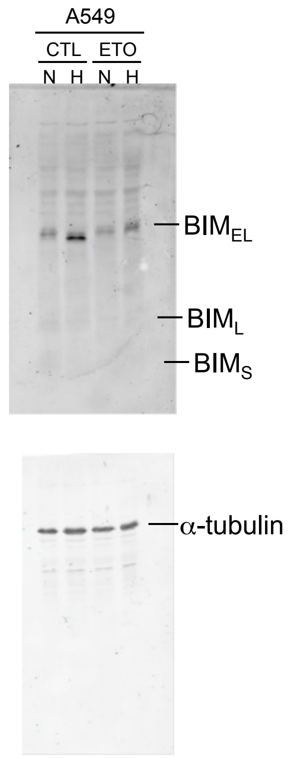

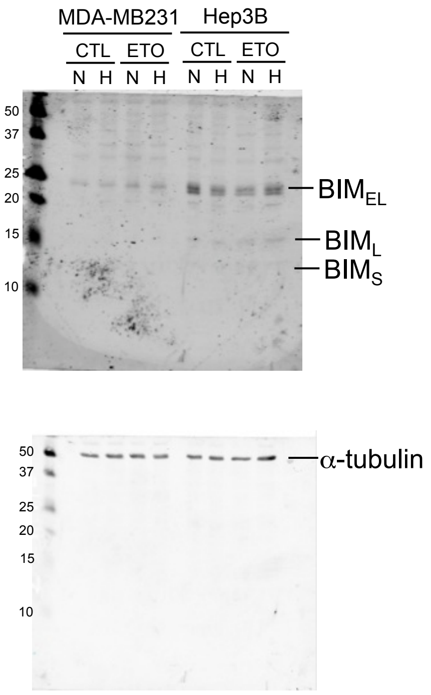

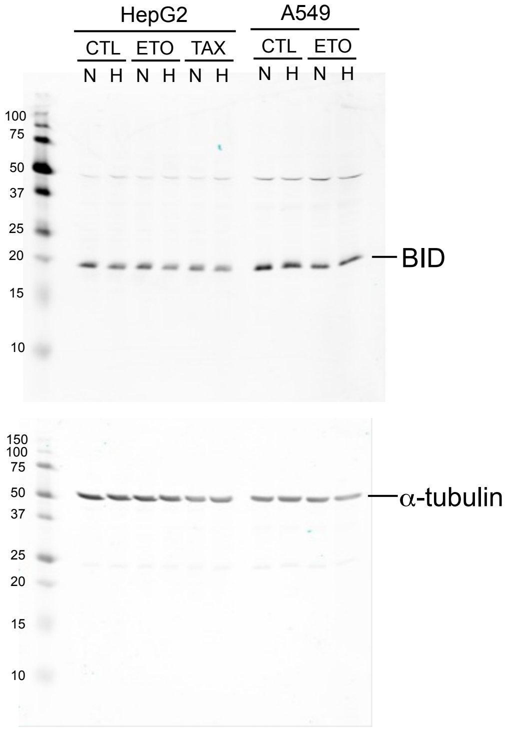

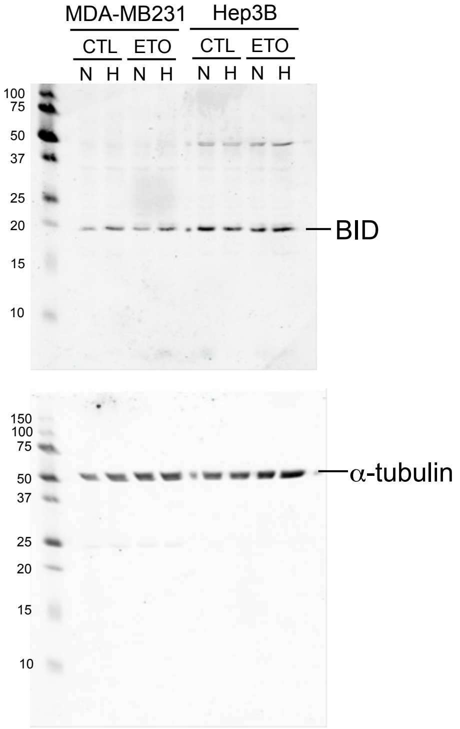

Western blots from figure 4

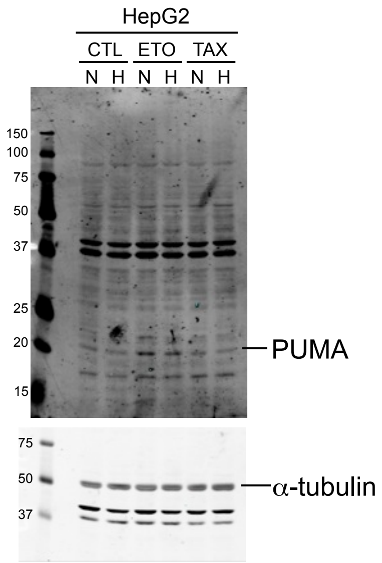

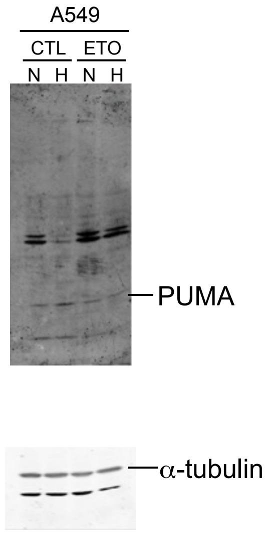

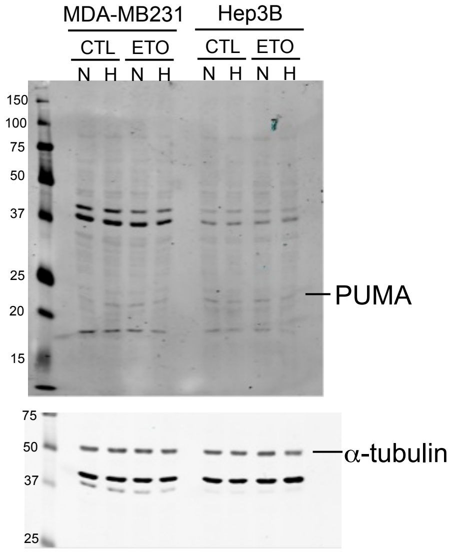

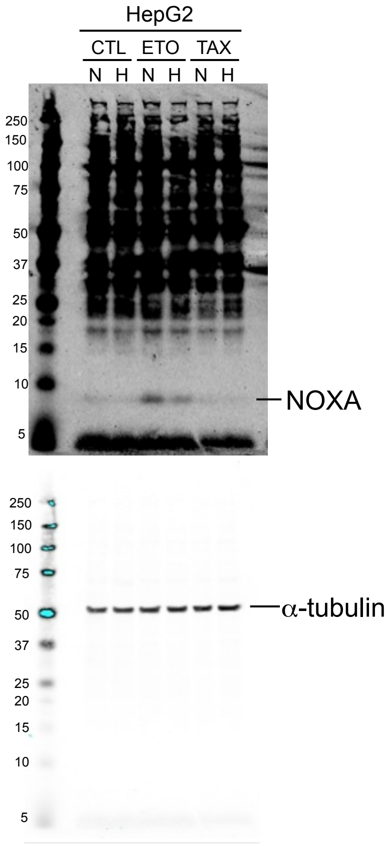

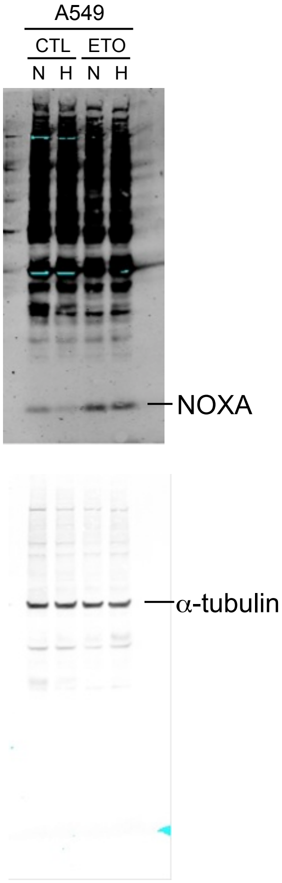

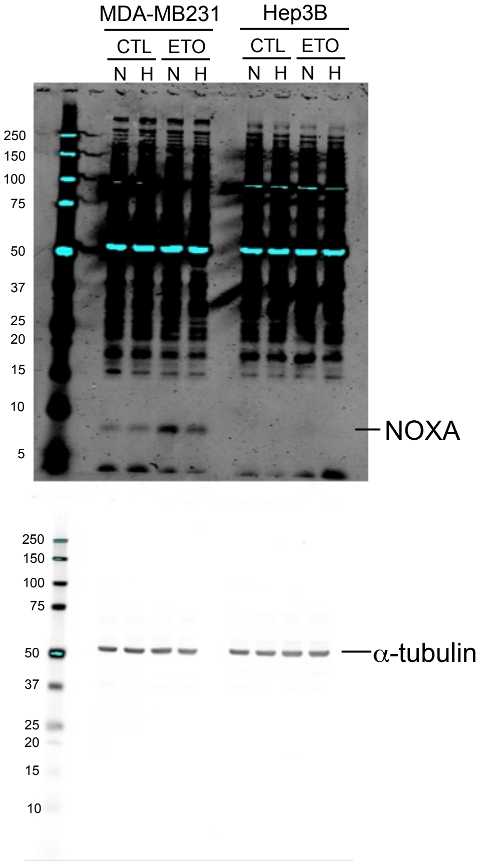

Western blots from figure 4

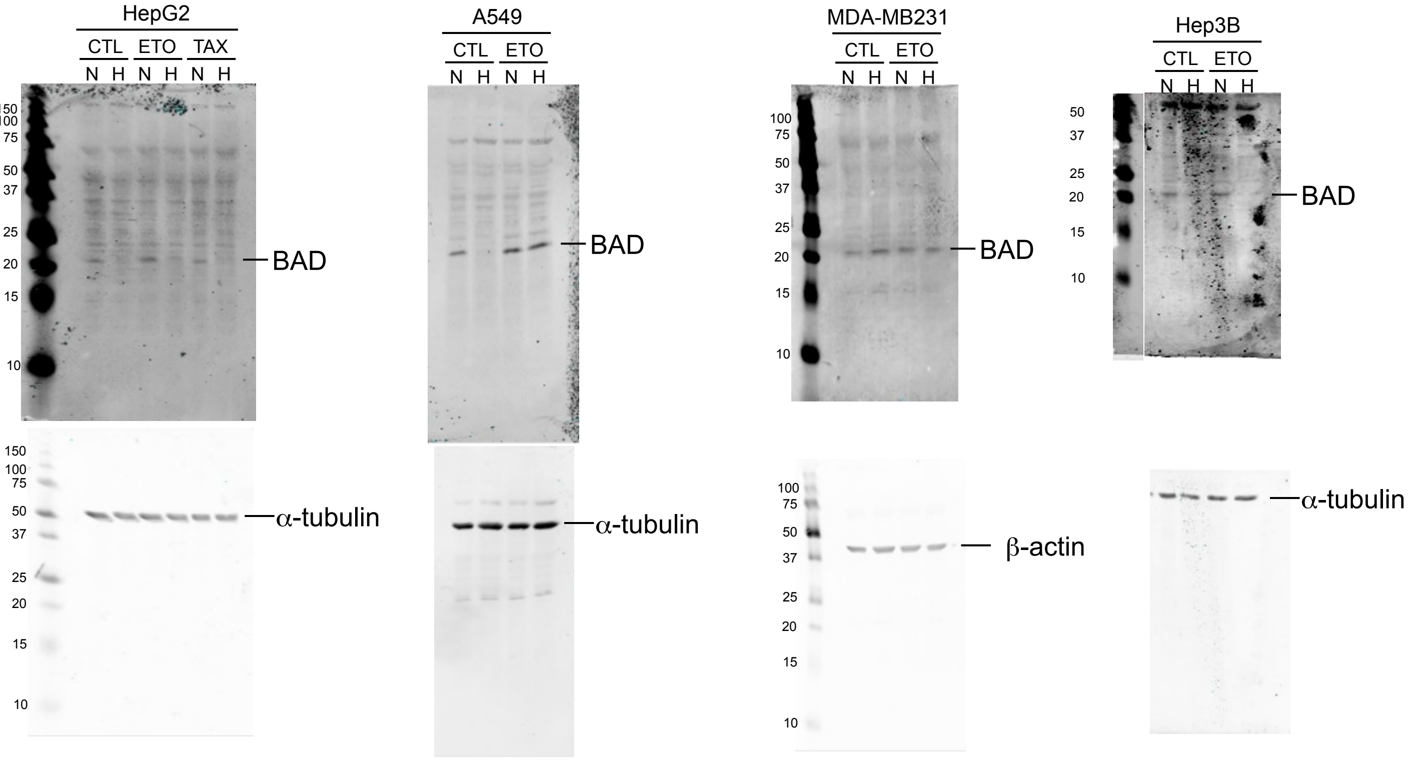

Western blots from figure 4

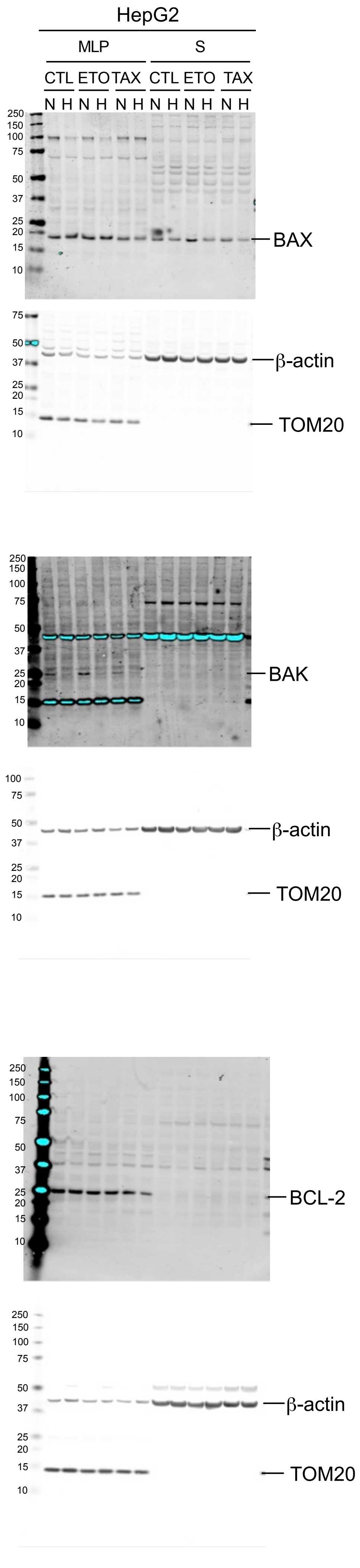

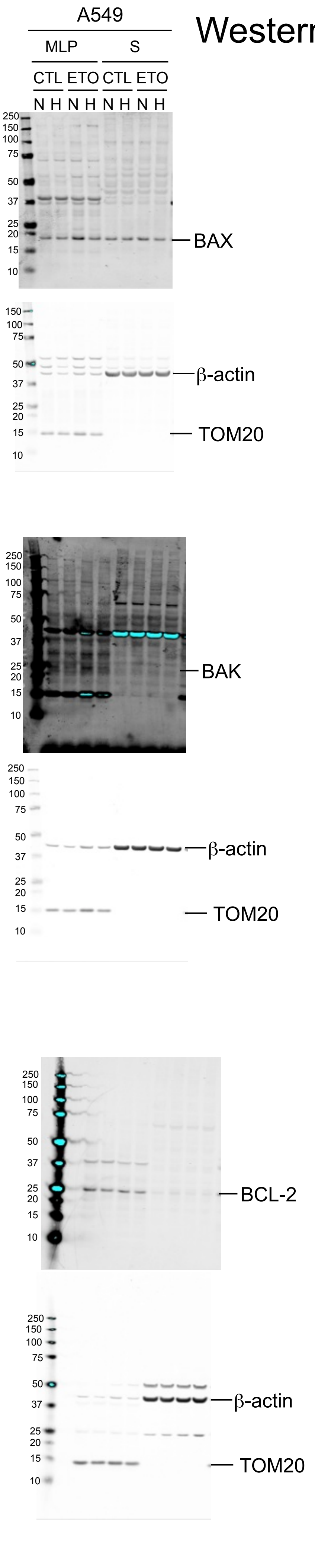

Western blots from figure 4

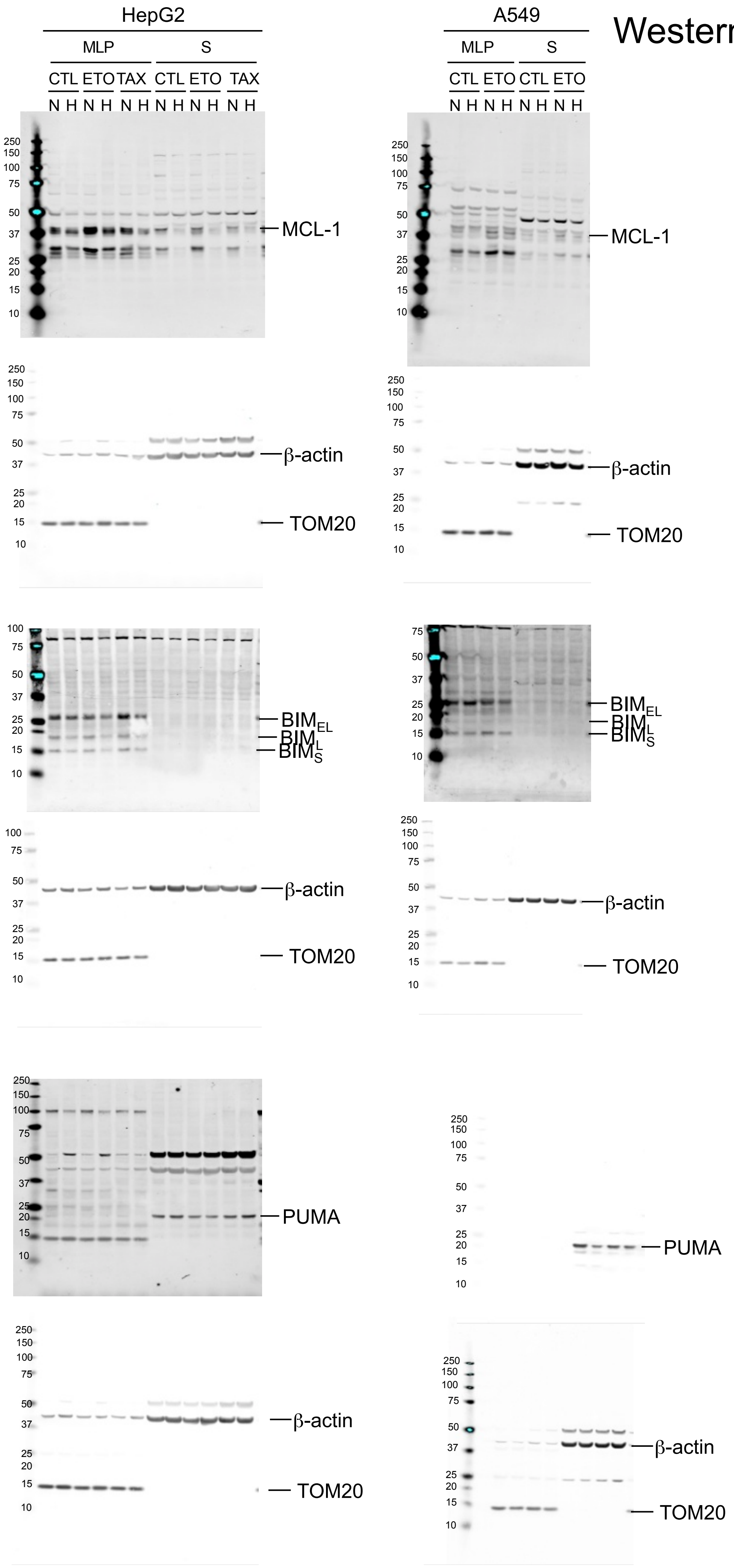

Western blots from figure 4

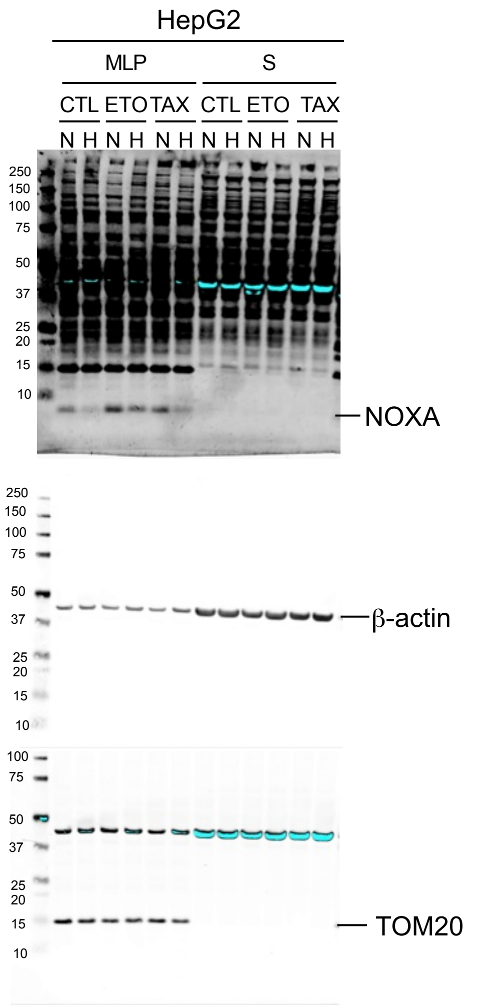

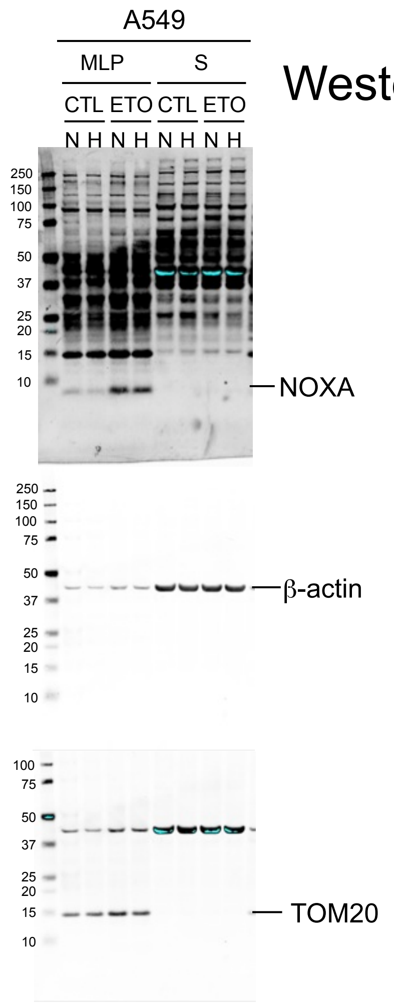

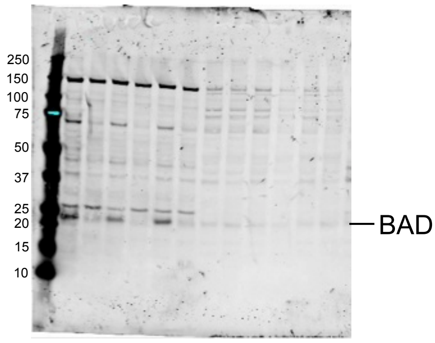

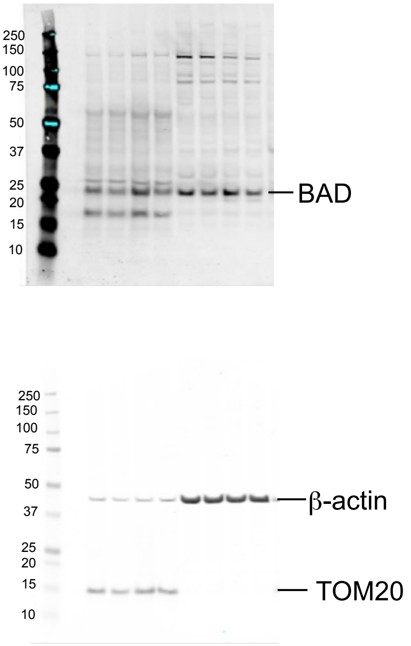

Western blots from figure 6

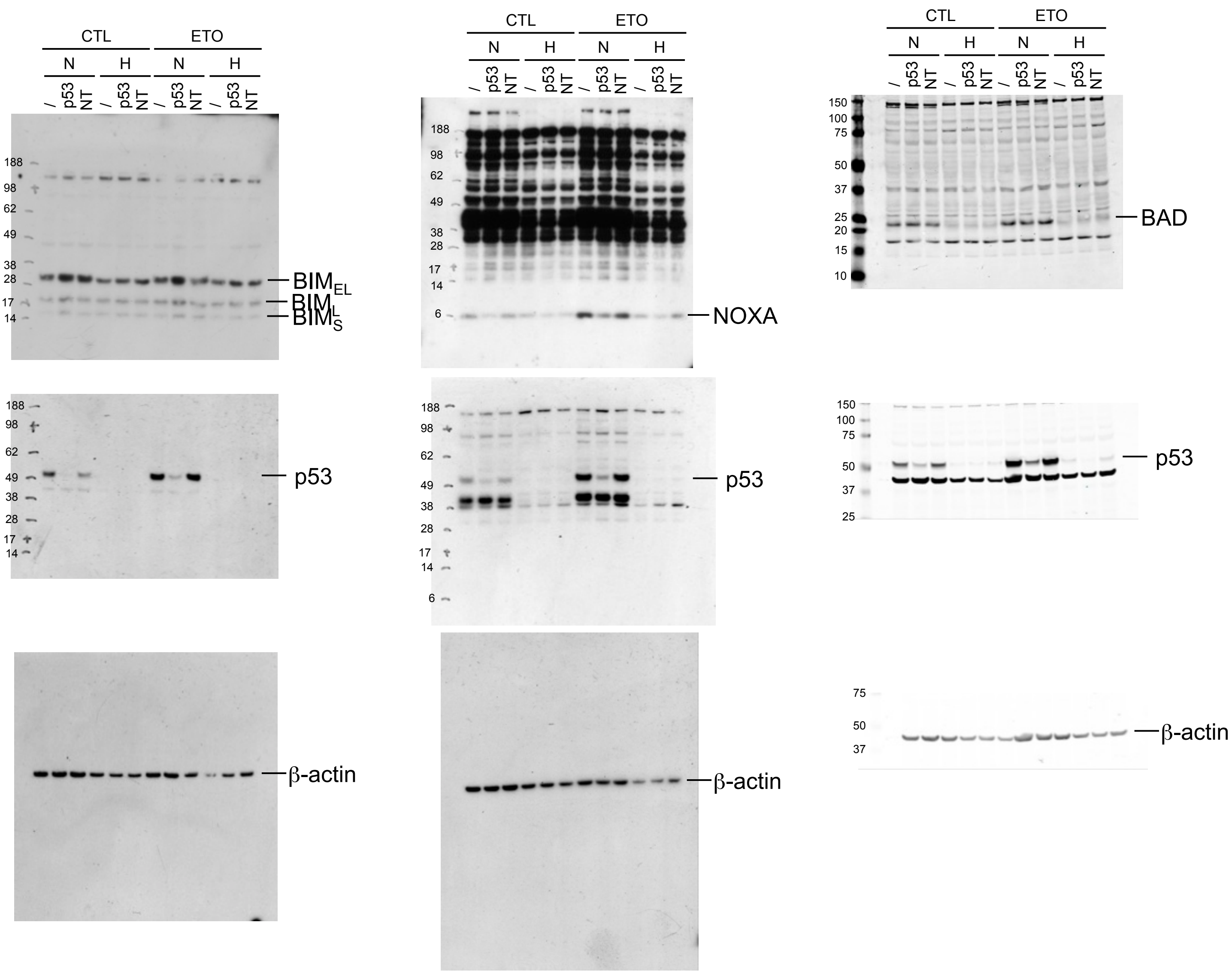

Western blots from supplementary figure 2

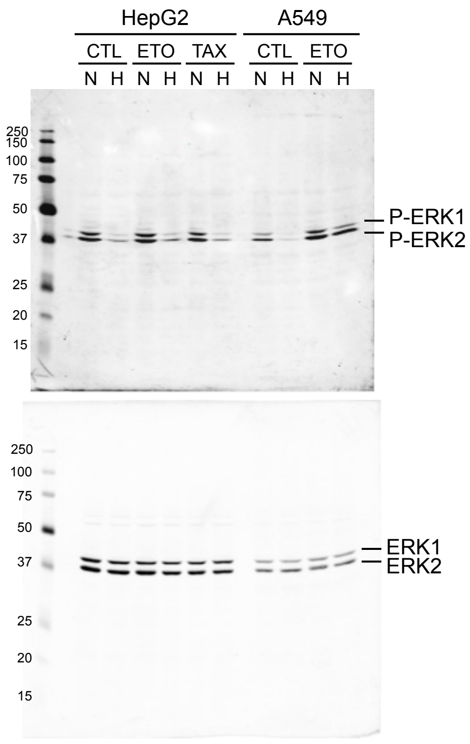

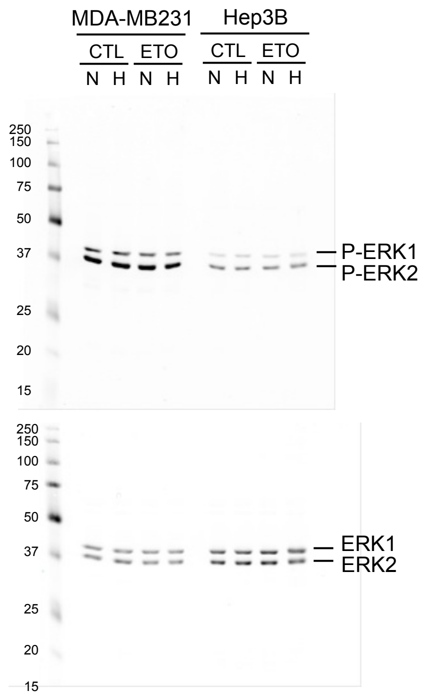

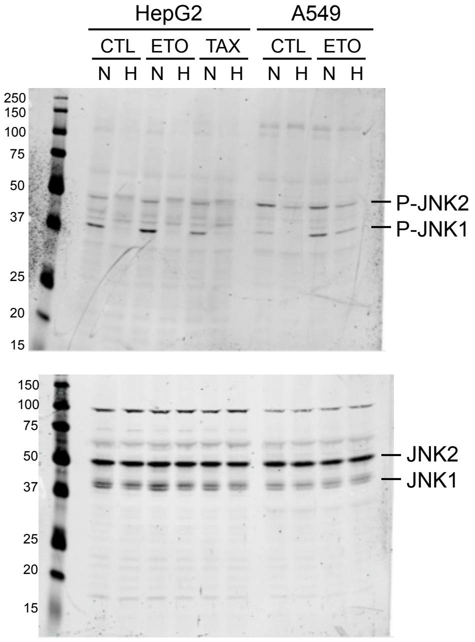

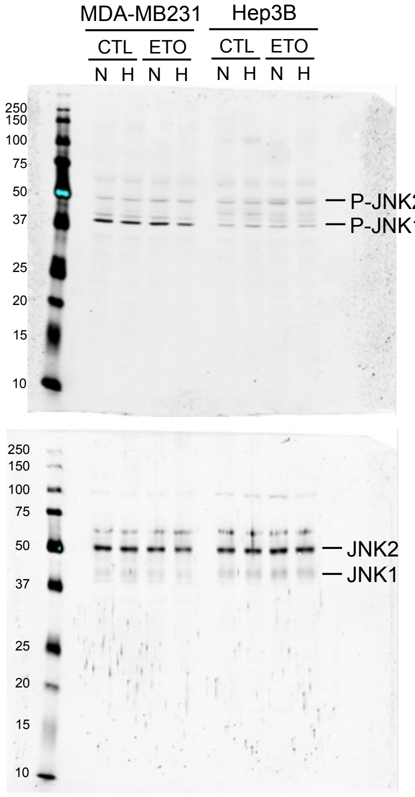

Western blots from supplementary figure 3

f

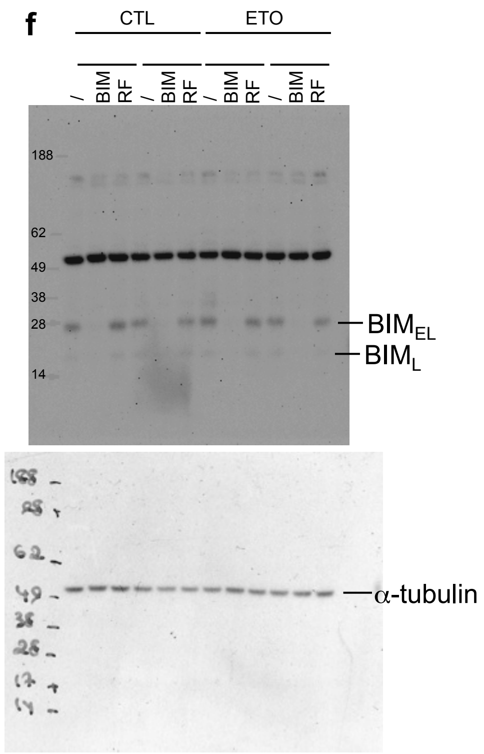

g

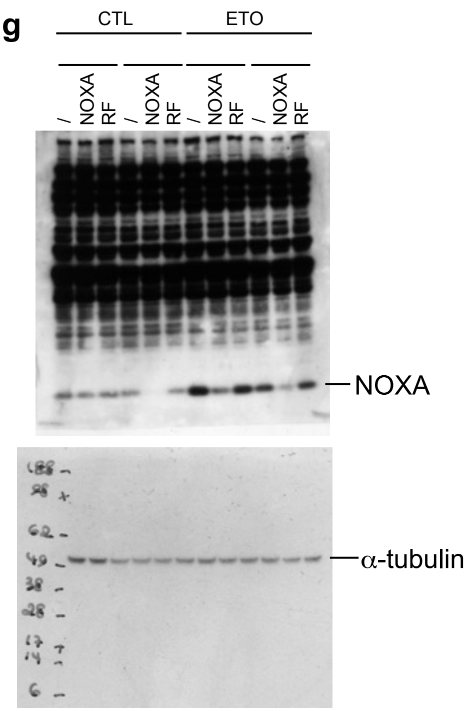

h

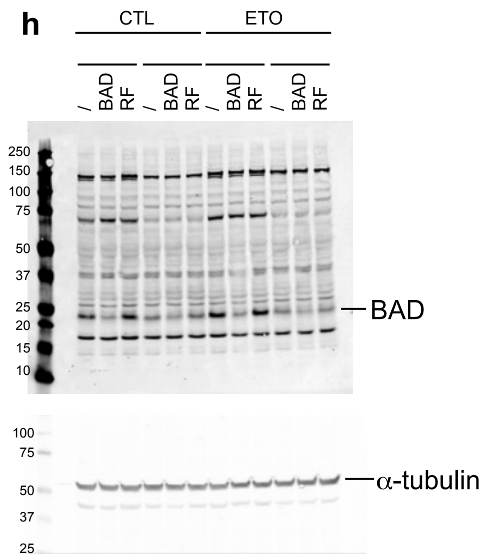

i

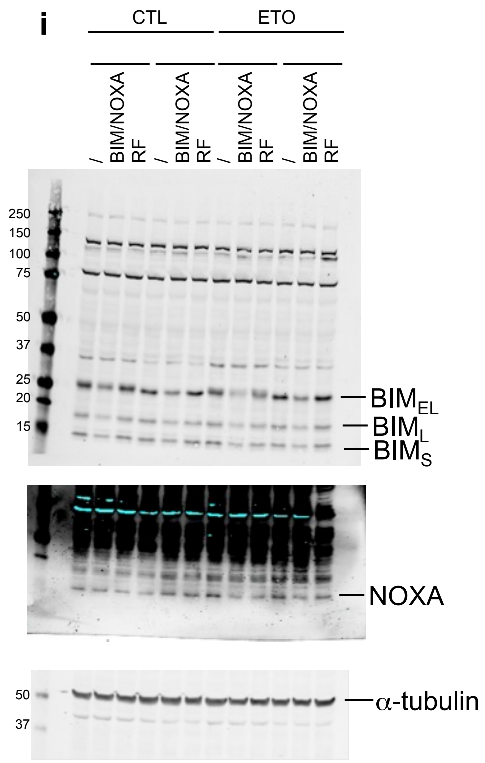

Supplement: Figure S1 — Uncropped western blots for Figures 2 , 3 , 6 and 8 and for Figures S2 and S3. (PDF) [file pone.0047519.s001.pdf]
