# Supplementary figures and images for: Hypoxia-Induced Modulation of Apoptosis and BCL-2 Family Proteins in Different Cancer Cell Types
Source: PLoS One. 2012 Nov 5;7(11):e47519. doi: 10.1371/journal.pone.0047519 (PMC3489905; doi:10.1371/journal.pone.0047519)

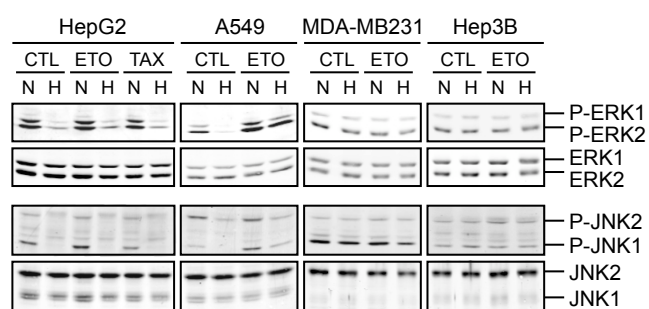

Supplementary figure 2

Supplement: Figure S2 — Effect of hypoxia, etoposide and paclitaxel on JNK and ERK phosphorylation. HepG2, A549, MDA-MB231 and Hep3B cells were incubated 16 hours under normoxia (N, 21% O2) or hypoxia (H, 1% O2) in the presence or not (CTL) of etoposide (ETO, 100 µM in Hep3B cells and 50 µM in the other cell types) or paclitaxel (TAX, 10 µM) in HepG2 cells. Proteins were detected in total cell extracts by western blotting, using specific antibodies. Uncropped western blots are presented in the supplementary figure 1. (PDF) [file pone.0047519.s002.pdf]

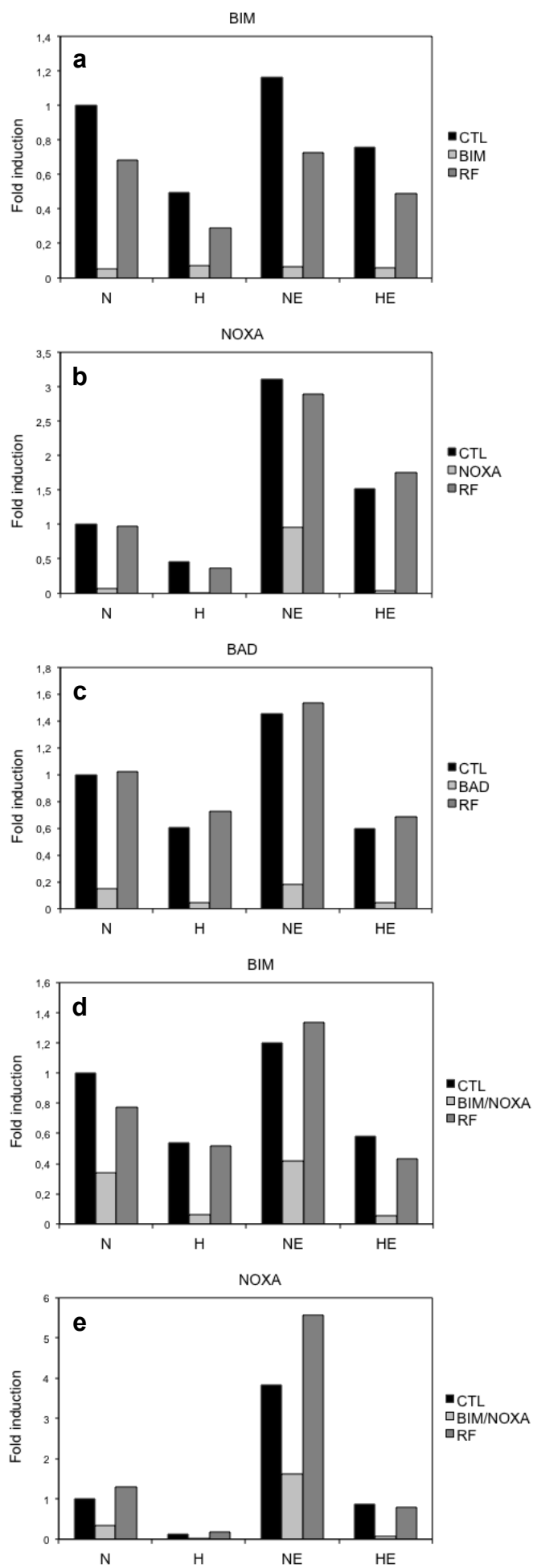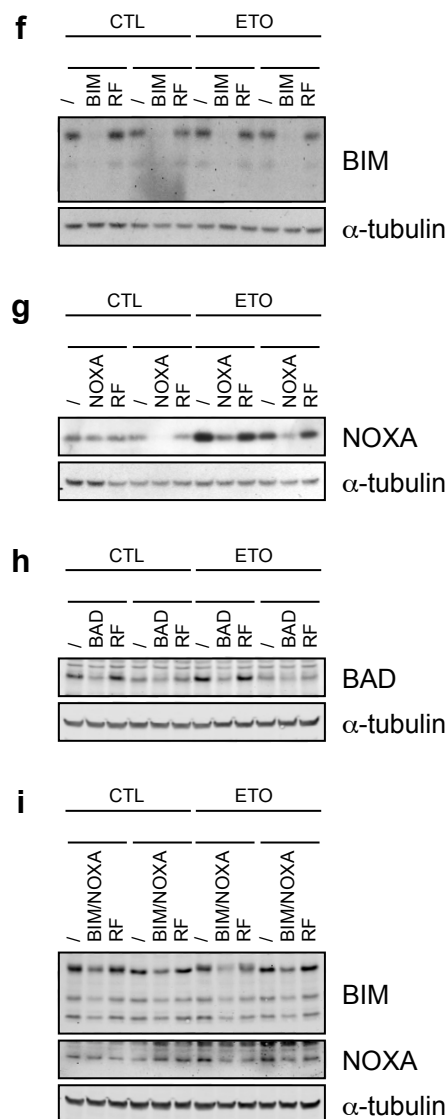

Supplementary figure 3

Supplement: Figure S3 — Effect of BH3-only proteins silencing on their mRNA and protein levels. HepG2 cells were transfected with 50 nM BIM (a, f), NOXA (b, g) or BAD (c, h) siRNAs, or 25 nM BIM combined with 25 nM NOXA siRNAs (d, e, i), or 50 nM RISC-free (RF) control siRNA or left untransfected (/) for 24 hours. 6 hours later (or 30 hours later for a and f), cells were incubated under normoxia (N, 21% O2) or hypoxia (H, 1% O2) with (ETO, E) or without (CTL) etoposide (50 µM) for 16 hours. (a, b, c, d, e) After incubation, total RNA was extracted, submitted to reverse transcription and to amplification in the presence of SYBR Green and specific primers (RT-PCR). RPL13A was used as housekeeping gene for data normalization. Data are given in fold induction (n = 1). (f, g, h, i) Proteins were detected in total cell extracts by western blotting, using specific antibodies. alpha-tubulin was used as loading control. Uncropped western blots are presented in Figure S1. (PDF) [file pone.0047519.s003.pdf]
