# Supplementary material for: Hypoxia-Induced Modulation of Apoptosis and BCL-2 Family Proteins in Different Cancer Cell Types
Source: PLoS One. 2012 Nov 5;7(11):e47519. doi: 10.1371/journal.pone.0047519 (PMC3489905; doi:10.1371/journal.pone.0047519)
Supplement: Table S1 — Sequences of the primers used for RT-real-time PCR. (PDF) [file pone.0047519.s004.pdf]

	forward primer	reverse primer
BAD	AGGATGAGTGACGAGTTTGTGGA	GGAGCTTTGCCGCATCTG
BAK	CTTCGTGGTCGACTTCATGCT	GGACCATTGCCCAAGTTCAG
BAX	TGTCGCCCTTTTCTACTTTGC	GTCCAGCCCATGATGGTTCT
BCL2L11 (BIM)	AAAAGACCAAATGGCAAAGCA	CTCTCCGCAGGCTGCAA
BIK	TGAAGAGGACCTGGACCCTATG	CCTGAGGCTCACGTCCATCT
BIRC3	GTCCCAGGGTGAGGCAAGA	TAAAGGCAGGGTCTCAGTATGCT
BNIP3	TTTGCTGGCCATCGGATT	ACCAAGTCAGACTCCAGTTCTTCA
CASP10	CCACAGCTTTACCTCCCTGAAG	CATTTCCACTTCGTCACATTATTG
CASP3	GAGGCCGACTTCTTGTATGCA	CAAAGCGACTGGATGAACCA
DEDD2	TGGCACCTCCAGCTCTTCA	CCGCTGCCGCTTGGT
LDHA	TGGCAGAGAGTATAATGAAGAATCTTAGG	GAAGACATCATCCTTTATTCCGTAAAG
MCL-1	AAACGGGACTGGCTAGTTAAACAA	TACTCCAGCAACACCTGCAAA
NALP1	GCTGGAAATACTCCCCAAGGA	CACACCAGAGTCTCATCTTTCTTGTC
PMAIP1 (NOXA)	CGCGCAAGAACGCTCAA	TGCCGGAAGTTCAGTTTGTCT
PUMA	ACGACCTCAACGCACAGTACG	AGGAGTCCCATGATGAGATTGTACA
RPL13A	GCCTACAAGAAAGTTTGCCTATCTG	TGAGCTGTTTCTTCTTCCGGTAGT
TRADD	GGGTCAGCCTGTAGTGAATCG	CACCTTGCGCCATTTGAGA

Supplementary table 1
