# Supplementary material for: Hypoxia-Induced Modulation of Apoptosis and BCL-2 Family Proteins in Different Cancer Cell Types
Source: PLoS One. 2012 Nov 5;7(11):e47519. doi: 10.1371/journal.pone.0047519 (PMC3489905; doi:10.1371/journal.pone.0047519)
Supplement: Table S2 — Effect of hypoxia, etoposide and paclitaxel on the mRNA expression level of genes involved in the apoptotic pathway. Results were obtained using “TLDA Human Apoptosis Panel” (Applied Biosystems) (TLDA). HepG2, A549, MDA-MB231 and Hep3B cells were incubated 16 hours under normoxia (N, 21% O2) or hypoxia (H, 1% O2) in the presence or not of etoposide (E, 100 µM in Hep3B cells and 50 µM in the other cell types) or paclitaxel (T, 10 µM) in HepG2 cells. After incubation, total RNA was extracted, submitted to reverse transcription and then to TLDA analysis. 18S was used as housekeeping gene for data normalization. Data are given in fold-induction. Grey cell notify that the Ct value was >35 and should therefore not be considered as quantitative. “-” non expressed or not possible to calculate a fold change because the mRNA is not expression in controle cells. Genes shown in bold are genes whose expression was validated by qRT-PCT. (PDF) [file pone.0047519.s005.pdf]

FI 18S	HepG2						A549				MDA-MB-231				Hep3B			
**Detector**	**N**	**H**	**NE**	**HE**	**NT**	**HT**	**N**	**H**	**NE**	**HE**	**N**	**H**	**NE**	**HE**	**N**	**H**	**NE**	**HE**
18S-Hs99999901_s1	1,00	1,00	1,00	1,00	1,00	1,00	1,00	1,00	1,00	1,00	1,00	1,00	1,00	1,00	1,00	1,00	1,00	1,00
ACTB-Hs99999903_m1	1,00	1,02	0,67	0,48	0,50	0,58	1,00	0,70	0,35	0,45	1,00	0,69	0,36	0,37	1,00	1,00	0,59	0,50
APAF1-Hs00559441_m1	1,00	0,73	2,14	1,74	0,73	0,77	1,00	0,87	1,49	1,05	1,00	1,20	0,87	1,09	1,00	0,96	0,85	1,14
BAD-Hs00188930_m1	1,00	0,64	1,32	0,93	1,62	1,01	1,00	0,59	0,47	0,43	1,00	0,54	1,18	0,87	1,00	0,75	-	1,14
**BAK1-Hs00832876_g1**	1,00	0,65	1,65	1,21	0,84	0,51	1,00	0,35	1,38	1,08	1,00	0,82	0,67	0,65	1,00	0,77	0,86	0,59
**BAX-Hs00751844_s1**	1,00	0,58	1,65	1,39	0,94	0,77	1,00	0,36	2,26	1,53	1,00	0,64	1,10	0,88	1,00	0,70	0,82	0,70
**BBC3-Hs00248075_m1**	1,00	0,35	1,09	1,03	0,92	0,69	1,00	0,56	2,06	2,56	1,00	0,74	0,96	0,99	1,00	0,57	0,87	0,82
BCAP31-Hs01036137_m1	1,00	0,94	1,13	0,74	0,87	0,94	1,00	0,65	0,53	0,73	1,00	0,91	0,91	0,88	1,00	1,08	0,89	0,88
BCL10-Hs00961847_m1	1,00	0,58	1,21	1,47	0,49	0,43	1,00	0,73	1,41	3,88	1,00	1,86	1,28	3,06	1,00	1,09	1,91	2,86
BCL2A1-Hs00187845_m1	1,00	0,74	1,09	1,02	0,49	0,40	1,00	1,92	10,22	9,48	1,00	2,38	0,82	5,33	-	-	-	-
BCL2-Hs00608023_m1	1,00	1,61	1,95	-	1,86	3,03	1,00	0,33	0,21	0,07	1,00	0,39	0,96	0,83	1,00	1,95	2,26	5,43
BCL2L10-Hs00368095_m1	-	-	-	-	-	-	-	-	-	-	-	-	-	-	-	-	-	-
**BCL2L11-Hs00708019_s1**	1,00	0,50	1,27	0,63	1,10	0,64	1,00	0,91	0,59	1,04	1,00	0,48	2,61	2,58	1,00	0,86	1,67	1,94
BCL2L13-Hs00209789_m1	1,00	0,60	1,09	0,76	1,44	1,08	1,00	0,42	0,52	0,30	1,00	0,67	0,63	0,51	1,00	0,84	0,62	0,71
BCL2L14-Hs00373302_m1	1,00	0,20	0,32	0,49	0,48	0,20	-	-	-	-	1,00	-	4,41	3,59	1,00	0,86	1,61	2,27
BCL2L1-Hs00169141_m1	1,00	0,82	0,75	1,15	1,21	1,69	1,00	0,82	0,50	0,52	1,00	0,92	0,28	0,24	1,00	1,60	0,23	0,34
BCL2L2-Hs00187848_m1	1,00	0,88	0,95	0,76	2,04	1,40	1,00	0,56	0,47	0,75	1,00	463,62	638,55	775,57	1,00	0,56	0,54	0,66
BCL3-Hs00180403_m1	1,00	1,17	0,84	1,18	1,10	1,02	1,00	0,90	0,53	0,52	1,00	0,52	0,34	0,41	1,00	1,22	0,47	0,45
BID-Hs00609632_m1	1,00	0,76	0,97	0,57	0,87	0,67	1,00	0,52	0,35	0,58	1,00	1,21	0,70	0,93	1,00	0,60	0,46	0,49
**BIK-Hs00154189_m1**	1,00	0,30	1,49	0,70	1,07	0,40	1,00	0,53	1,40	1,42	1,00	0,65	4,25	2,95	1,00	0,28	0,62	0,44
BIRC1-Hs01847653_s1	1,00	1,92	0,44	0,45	2,86	2,31	1,00	-	2,88	11,54	1,00	0,61	0,02	0,14	1,00	1,40	1,57	2,28
BIRC2-Hs00236911_m1	1,00	0,69	0,55	0,57	1,06	0,77	1,00	0,81	0,69	0,60	1,00	1,72	1,17	1,27	1,00	1,21	0,51	1,01
**BIRC3-Hs00985031_g1**	1,00	1,21	0,54	0,98	0,50	0,55	1,00	0,56	0,78	0,65	1,00	1,07	1,10	2,14	1,00	2,01	0,58	1,03
BIRC4-Hs00745222_s1	1,00	0,68	0,85	0,66	0,67	0,60	1,00	0,64	0,80	0,51	1,00	0,79	0,60	0,62	1,00	0,88	0,65	0,67
BIRC5-Hs00977611_g1	1,00	0,50	0,83	0,49	1,20	0,77	1,00	0,47	0,08	0,05	1,00	0,65	0,99	0,70	1,00	0,83	1,61	1,27
BIRC6-Hs00212288_m1	1,00	0,69	0,85	0,62	0,96	0,85	1,00	0,68	0,43	0,59	1,00	0,91	0,33	0,31	1,00	0,89	0,24	0,21
BIRC7-Hs00223384_m1	1,00	6,51	7,77	13,91	0,76	7,52	1,00	3,20	0,98	1,95	-	-	-	-	-	-	-	-
BIRC8-Hs01057786_s1	1,00	1,79	0,34	-	1,28	1,92	1,00	-	0,50	0,56	1,00	0,60	0,07	0,22	1,00	0,45	1,21	0,21
BNIP3-Hs00969291_m1	1,00	5,74	0,82	4,97	0,88	6,47	1,00	3,88	0,89	5,19	1,00	13,12	1,56	9,31	1,00	12,23	1,35	10,35
BNIP3L-Hs00188949_m1	1,00	12,80	2,42	13,15	1,02	11,74	1,00	3,04	0,46	2,58	1,00	4,38	0,74	1,89	1,00	14,48	1,93	15,23
BOK-Hs00261296_m1	1,00	0,95	0,74	0,96	0,78	1,17	1,00	0,97	0,32	0,99	1,00	0,63	0,94	1,38	1,00	0,84	0,65	0,90
CARD15-Hs00223394_m1	1,00	3,03	6,32	3,03	1,73	2,50	1,00	-	0,65	1,51	-	-	-	-	-	-	-	-
CARD4-Hs00196075_m1	1,00	0,30	0,97	0,45	0,73	0,44	1,00	0,66	0,58	0,54	1,00	0,54	1,28	1,19	1,00	1,13	2,79	1,53
CARD6-Hs00261581_m1	-	-	-	-	-	-	1,00	0,38	2,59	12,45	1,00	1,12	2,38	4,12	-	-	-	-
CARD9-Hs00364485_m1	1,00	0,70	0,73	0,13	1,74	0,88	-	-	-	-	1,00	-	0,41	1,35	1,00	0,45	1,58	0,87
**CASP10-Hs01017902_m1**	1,00	0,40	1,05	0,59	0,61	0,54	1,00	0,45	1,34	1,37	1,00	0,56	1,21	0,89	1,00	1,27	1,79	1,72
CASP14-Hs00201637_m1	-	-	-	-	-	-	-	-	-	-	-	-	-	-	-	-	-	-
CASP1-Hs00354836_m1	-	-	-	-	-	-	1,00	0,30	6,72	2,24	1,00	0,21	2,67	1,57	1,00	0,32	0,45	0,48
CASP2-Hs00892481_m1	1,00	0,43	0,89	0,75	0,82	0,45	1,00	0,43	0,30	0,19	1,00	0,69	1,14	1,00	1,00	0,83	1,24	1,24
**CASP3-Hs00263337_m1**	1,00	0,43	0,88	0,39	0,69	0,34	1,00	0,34	0,56	0,77	1,00	0,70	1,58	1,73	1,00	0,59	1,21	1,43
CASP4-Hs01031947_m1	1,00	0,61	0,94	0,46	0,49	0,49	1,00	0,60	0,83	1,18	1,00	0,75	2,16	2,38	1,00	1,35	1,13	1,56
CASP5-Hs00362072_m1	1,00	-	-	-	-	-	-	-	-	-	-	-	-	-	-	-	-	-
CASP6-Hs00154250_m1	1,00	0,43	1,88	0,93	0,84	0,53	1,00	0,60	0,60	0,74	1,00	0,64	0,90	1,11	1,00	0,64	0,77	1,15
CASP7-Hs00169152_m1	1,00	0,43	1,05	0,74	0,85	0,48	1,00	0,71	0,81	0,56	1,00	0,73	1,82	1,59	1,00	0,70	1,91	1,90
CASP8AP2-Hs01594281_m1	1,00	0,73	0,71	0,57	0,82	0,51	1,00	0,47	0,36	0,49	1,00	0,62	1,47	1,63	1,00	0,89	1,06	1,32
CASP8-Hs01018151_m1	1,00	0,43	1,00	0,60	0,80	0,38	1,00	0,65	0,88	0,52	1,00	0,80	0,78	0,84	1,00	0,93	1,30	1,46
CASP9-Hs00154260_m1	-	-	-	-	-	-	-	-	-	-	1,00	0,59	-	1,16	1,00	0,74	1,84	1,46
CFLAR-Hs00153439_m1	1,00	0,61	1,19	0,77	0,67	0,60	1,00	0,70	0,71	1,19	1,00	0,87	0,94	0,99	1,00	0,84	0,60	0,68
CHUK-Hs00989502_m1	1,00	0,48	1,17	0,78	0,65	0,56	1,00	0,44	0,74	0,79	1,00	0,50	1,50	1,40	1,00	0,60	0,84	0,76
CRADD-Hs01011159_g1	1,00	1,01	1,35	1,59	1,06	1,45	1,00	0,75	0,60	0,84	1,00	1,11	0,28	0,36	1,00	0,89	0,50	0,50
DAPK1-Hs00234480_m1	1,00	0,59	0,92	0,63	0,74	0,61	1,00	1,75	0,37	1,40	-	-	-	-	1,00	1,79	0,22	0,32
**DEDD2-Hs00370206_m1**	1,00	1,08	2,66	1,99	1,14	0,96	1,00	0,82	1,31	1,29	1,00	0,97	1,54	1,88	1,00	1,59	2,94	2,51
DEDD-Hs00172768_m1	1,00	0,63	1,73	1,28	1,05	0,65	1,00	0,64	0,36	0,45	1,00	0,71	0,87	0,97	1,00	0,80	1,45	1,46
DIABLO-Hs00219876_m1	1,00	0,89	1,01	0,92	0,91	0,79	1,00	0,73	0,66	0,86	1,00	0,84	1,27	1,16	1,00	0,86	1,18	1,50
ESRRBL1-Hs00215973_m1	1,00	0,45	0,51	0,48	1,26	0,78	1,00	0,46	1,30	0,95	1,00	0,59	0,83	0,68	1,00	-	18,19	11,03
FADD-Hs00538709_m1	1,00	0,64	1,05	0,85	0,83	0,80	1,00	0,55	0,37	0,57	1,00	0,49	0,46	0,65	1,00	0,60	0,84	0,86
FAS-Hs00236330_m1	1,00	0,28	3,01	1,20	1,15	0,39	1,00	0,37	4,32	2,92	1,00	1,16	1,26	1,58	-	-	-	-
FASLG-Hs00181225_m1	-	-	-	-	-	-	-	-	-	-	1,00	0,24	0,28	0,89	1,00	1,01	8,35	3,69
GAPDH-Hs99999905_m1	1,00	1,84	0,91	1,96	1,00	2,23	1,00	1,43	0,48	1,22	1,00	1,79	0,91	1,53	1,00	2,83	1,25	3,22
HIP1-Hs00193477_m1	1,00	0,57	1,86	0,83	2,02	1,31	1,00	1,06	0,25	0,42	1,00	0,56	0,71	0,81	1,00	1,10	0,53	0,56
HRK-Hs00705213_s1	1,00	0,51	0,12	0,12	0,12	0,12	0,12	0,12	0,12	0,12	1,00	0,40	0,12	0,32	1,00	1,10	0,49	0,70
HTRA2-Hs00376860_g1	1,00	0,40	0,86	0,45	0,58	0,50	1,00	0,49	0,68	0,62	1,00	0,53	1,04	0,92	1,00	0,58	0,91	0,66
ICEBERG-Hs01043258_m1	-	-	-	-	-	-	-	-	-	-	-	-	-	-	-	-	-	-
IKBKB-Hs00395088_m1	1,00	0,59	1,31	0,71	0,84	0,53	1,00	0,52	0,68	0,57	1,00	0,92	0,53	0,60	1,00	0,76	0,66	0,34
IKBKE-Hs01063858_m1	1,00	0,74	0,55	0,45	1,11	1,01	1,00	0,81	0,45	0,86	1,00	1,05	0,70	0,90	1,00	0,96	0,42	0,38
IKBKG-Hs00175318_m1	1,00	0,57	1,02	0,72	1,14	1,28	1,00	0,52	0,42	0,56	1,00	0,87	0,92	0,97	1,00	1,53	0,72	1,20
LRDD-Hs00388035_m1	1,00	0,30	3,16	1,24	1,07	0,56	1,00	0,50	3,69	2,75	1,00	0,82	2,46	1,90	1,00	1,07	1,55	1,60
LTA-Hs99999086_m1	-	-	-	-	-	-	1,00	1,08	4,38	7,52	1,00	1,05	0,81	0,89	1,00	4,81	99,96	89,30
LTB-Hs00242739_m1	1,00	-	-	0,70	-	0,59	1,00	1,72	0,20	0,56	1,00	0,56	0,80	1,25	1,00	4,97	0,17	0,24
**MCL1-Hs00172036_m1**	1,00	1,27	1,13	2,22	0,82	1,39	1,00	1,11	0,93	1,00	1,00	1,89	1,77	1,86	1,00	1,03	1,58	1,37
**NALP1-Hs00248187_m1**	1,00	0,18	4,90	2,38	0,79	-	1,00	1,04	9,51	15,58	1,00	1,14	0,89	1,22	1,00	1,94	1,67	1,75
NFKB1-Hs00765730_m1	1,00	0,76	0,54	0,67	0,97	0,84	1,00	0,52	0,66	0,56	1,00	0,86	0,50	0,54	1,00	1,07	0,33	0,44
NFKB2-Hs00174517_m1	1,00	0,66	0,74	0,71	0,80	0,67	1,00	0,63	0,56	0,51	1,00	1,02	1,94	2,35	1,00	1,42	0,99	0,99
NFKBIA-Hs00153283_m1	1,00	0,99	1,06	1,32	0,82	0,83	1,00	1,16	0,72	1,43	1,00	0,76	0,73	0,87	1,00	1,07	0,90	0,90
NFKBIB-Hs00182115_m1	1,00	0,56	0,69	0,45	0,82	0,48	1,00	0,39	0,92	0,64	1,00	0,54	1,91	1,52	1,00	0,83	0,56	0,70
NFKBIE-Hs00234431_m1	1,00	0,88	0,73	1,19	0,74	0,70	1,00	0,61	0,72	0,82	1,00	0,77	1,64	1,89	1,00	1,18	0,63	0,56
NFKBIZ-Hs00230071_m1	1,00	0,59	0,80	0,42	0,44	0,38	1,00	0,57	1,62	1,19	1,00	0,89	0,98	0,57	1,00	0,38	0,94	0,52
PEA15-Hs00269428_m1	1,00	0,50	0,89	0,66	0,86	0,43	1,00	0,36	0,45	0,49	1,00	0,73	1,45	1,37	1,00	0,86	1,31	1,56
**PMAIP1-Hs00560402_m1**	1,00	0,45	2,70	1,27	0,84	0,48	1,00	0,25	1,92	1,22	1,00	0,93	2,41	2,04	1,00	1,84	7,98	13,95
PYCARD-Hs00203118_m1	1,00	0,51	1,78	1,29	1,05	1,02	1,00	1,06	0,65	1,18	-	-	-	-	1,00	0,81	0,59	0,40
RELA-Hs00153294_m1	1,00	0,95	1,27	1,47	0,81	0,99	1,00	0,75	0,46	0,60	1,00	0,97	1,47	1,54	1,00	1,30	1,58	1,72
RELB-Hs00232399_m1	1,00	0,61	1,01	0,79	0,67	0,39	1,00	0,64	1,05	0,92	1,00	0,86	2,16	3,22	1,00	1,11	0,99	0,54
REL-Hs00968436_m1	1,00	0,71	0,68	0,64	0,87	0,95	1,00	0,54	0,50	0,73	1,00	1,24	1,55	2,13	1,00	0,85	0,97	1,26
RIPK1-Hs00169407_m1	1,00	0,42	1,41	0,94	0,97	0,59	1,00	0,81	0,64	0,95	1,00	0,76	1,08	1,21	1,00	1,11	1,35	2,13
RIPK2-Hs01572688_m1	1,00	0,50	0,61	0,34	0,67	0,34	1,00	0,86	0,53	0,92	1,00	0,78	2,07	2,46	1,00	0,83	1,43	1,97
TA-NFKBH-Hs01076336_m1	1,00	0,35	0,27	0,17	0,54	0,38	1,00	0,67	0,37	0,43	1,00	1,19	1,76	1,29	1,00	0,85	0,95	1,50
TBK1-Hs00179410_m1	1,00	0,67	0,89	0,94	0,68	0,83	1,00	0,38	1,20	0,82	1,00	0,65	1,03	0,77	1,00	0,90	0,90	1,10
TNF-Hs00174128_m1	1,00	1,07	-	0,41	0,61	0,38	1,00	0,65	0,93	1,54	-	-	-	-	-	-	-	-
TNFRSF10A-Hs00269492_m1	1,00	0,45	1,67	0,94	1,21	0,60	1,00	0,56	1,36	0,87	1,00	0,75	0,95	0,72	1,00	0,97	0,45	0,57
TNFRSF10B-Hs00366272_m1	1,00	0,66	0,73	0,51	0,79	0,62	1,00	0,57	0,66	1,06	1,00	0,80	0,60	0,61	1,00	1,06	0,59	0,96
TNFRSF1A-Hs01042313_m1	1,00	0,74	0,93	0,87	0,85	1,01	1,00	0,68	0,44	0,69	1,00	1,29	0,53	0,53	1,00	1,18	0,91	1,32
TNFRSF1B-Hs00153550_m1	-	-	-	-	-	-	1,00	4,42	1,01	0,76	1,00	0,39	0,60	0,51	1,00	1,04	2,17	5,68
TNFRSF21-Hs00205419_m1	1,00	0,56	0,92	0,38	1,10	0,51	1,00	0,45	0,69	0,42	1,00	0,42	0,34	0,21	1,00	0,46	0,27	0,27
TNFRSF25-Hs00980365_g1	1,00	0,43	1,16	0,42	0,65	0,80	1,00	2,57	1,22	2,37	1,00	0,56	1,02	0,21	1,00	1,83	0,94	0,96
TNFSF10-Hs00234356_m1	1,00	0,80	2,82	1,83	0,85	1,07	1,00	2,22	1,77	2,16	1,00	2,44	2,09	2,31	1,00	1,19	4,05	5,33
**TRADD-Hs00601065_g1**	1,00	0,53	1,68	0,97	0,59	0,77	1,00	1,53	0,54	1,37	1,00	1,24	0,71	1,07	1,00	1,10	0,57	0,93

Supplementary table 2
