# Supplementary material for: Hypoxia-Induced Modulation of Apoptosis and BCL-2 Family Proteins in Different Cancer Cell Types
Source: PLoS One. 2012 Nov 5;7(11):e47519. doi: 10.1371/journal.pone.0047519 (PMC3489905; doi:10.1371/journal.pone.0047519)
Supplement: Table S3 — Effect of hypoxia, etoposide and paclitaxel on the mRNA expression level of genes involved in the apoptotic pathway. Comparison of results obtained using “TLDA Human Apoptosis Panel” (Applied Biosystems) (TLDA) and single real-time RT-PCR reactions (RT-PCR). HepG2, A549, MDA-MB231 and Hep3B cells were incubated 16 hours under normoxia (N, 21% O2) or hypoxia (H, 1% O2) in the presence or not of etoposide (E, 100 µM in Hep3B cells and 50 µM in the other cell types) or paclitaxel (T, 10 µM) in HepG2 cells. After incubation, total RNA was extracted, submitted to reverse transcription and then to TLDA analysis or to amplification in the presence of SYBR Green and specific primers (RT-PCR). 18S and RPL13A were used as house keeping genes for data normalization of TLDA and single real-time RT-PCR reactions respectively. Data are given in fold-induction as the mean ±1 SD (n = 3) is provided for single real-time RT-PCR reactions. Refer to Table S2 for complete TLDA results. Grey cell notify that the Ct value was >35 and should therefore not be considered as quantitative. “-” non expressed or not possible to calculate a fold change because the mRNA is not expression in control cells. Genes shown in bold are genes whose expression was assessed at the protein level by western blot analyses. (PDF) [file pone.0047519.s006.pdf]

		HepG2						A549				MDA-MB231				Hep3B			
		N	H	NE	HE	NT	HT	N	H	NE	HE	N	H	NE	HE	N	H	NE	HE
**BAK**	TLDA	1,00	0,65	1,65	1,21	0,84	0,51	1,00	0,35	1,38	1,08	1,00	0,82	0,67	0,65	1,00	0,77	0,86	0,59
	RT-PCR	1,00 ± 0,15	0,43 ± 0,12	1,78 ± 0,46	1,25 ± 0,26	0,57 ± 0,30	0,41 ± 0,10	1,00 ± 0,04	0,43 ± 0,02	2,11 ± 0,29	1,39 ± 0,28	1,00 ± 0,17	0,61 ± 0,08	1,41 ± 0,21	0,96 ± 0,22	1,00 ± 0,23	0,81 ± 0,13	1,22 ± 0,25	0,86 ± 0,16
**BAX**	TLDA	1,00	0,58	1,65	1,39	0,94	0,77	1,00	0,36	2,26	1,53	1,00	0,64	1,10	0,88	1,00	0,70	0,82	0,70
	RT-PCR	1,00 ± 0,13	0,73 ± 0,11	1,43 ± 0,33	1,15 ± 0,26	0,91 ± 0,17	0,70 ± 0,17	1,00 ± 0,11	0,54 ± 0,14	3,02 ± 0,56	2,20 ± 0,58	1,00 ± 0,35	0,69 ± 0,24	1,00 ± 0,34	0,85 ± 0,30	1,00 ± 0,44	0,70 ± 0,11	0,93 ± 0,42	0,55 ± 0,15
**BBC3 = PUMA**	TLDA	1,00	0,35	1,09	1,03	0,92	0,69	1,00	0,56	2,06	2,56	1,00	0,74	0,96	0,99	1,00	0,57	0,87	0,82
	RT-PCR	1,00 ± 0,24	0,77 ± 0,30	2,29 ± 0,67	2,20 ± 0,39	0,94 ± 0,14	0,90 ± 0,28	1,00 ± 0,44	0,51 ± 0,14	1,93 ± 1,57	1,95 ± 0,22	1,00 ± 0,21	0,76 ± 0,10	1,00 ± 0,33	0,96 ± 0,49	1,00 ± 0,41	1,06 ± 0,27	1,08 ± 0,40	1,01 ± 0,22
**BCL2L11= BIM**	TLDA	1,00	0,50	1,27	0,63	1,10	0,64	1,00	0,91	0,59	1,04	1,00	0,48	2,61	2,58	1,00	0,86	1,67	1,94
	RT-PCR	1,00 ± 0,48	0,54 ± 0,20	1,03 ± 0,48	0,70 ± 0,40	0,94 ± 0,43	0,50 ± 0,18	1,00 ± 0,64	0,77 ± 0,43	1,44 ± 0,55	0,98 ± 0,34	1,00 ± 0,38	0,77 ± 0,21	3,80 ± 0,77	2,28 ± 0,66	1,00 ± 0,14	0,90 ± 0,23	2,12 ± 1,08	1,35 ± 0,53
BIK	TLDA	1,00	0,30	1,49	0,70	1,07	0,40	1,00	0,53	1,40	1,42	1,00	0,65	4,25	2,95	1,00	0,28	0,62	0,44
	RT-PCR	1,00 ± 1,31	0,27 ± 0,37	1,16 ± 1,16	0,84 ± 0,89	0,89 ± 1,18	0,37 ± 0,51	1,00 ± 0,56	0,40 ± 0,29	3,73 ± 2,17	3,17 ± 2,14	1,00 ± 0,27	0,68 ± 0,15	6,78 ± 0,73	3,83 ± 1,02	1,00 ± 0,24	0,33 ± 0,16	1,40 ± 0,27	0,90 ± 0,23
BIRC3	TLDA	1,00	1,21	0,54	0,98	0,50	0,55	1,00	0,56	0,78	0,65	1,00	1,07	1,10	2,14	1,00	2,01	0,58	1,03
	RT-PCR	1,00 ± 0,32	1,29 ± 0,34	0,51 ± 0,19	0,57 ± 0,08	0,69 ± 0,02	0,60 ± 0,54	1,00 ± 0,79	0,62 ± 0,20	0,72 ± 0,39	0,68 ± 0,06	1,00 ± 0,26	0,65 ± 0,11	0,74 ± 0,02	0,75 ± 0,20	1,00 ± 0,18	2,34 ± 0,46	0,86 ± 0,15	1,47 ± 0,63
CASP10	TLDA	1,00	0,40	1,05	0,59	0,61	0,54	1,00	0,45	1,34	1,37	1,00	0,56	1,21	0,89	1,00	1,27	1,79	1,72
	RT-PCR	1,00 ± 0,66	0,45 ± 0,21	1,20 ± 0,59	0,69 ± 0,42	0,64 ± 0,57	0,45 ± 0,16	1,00 ± 0,06	0,64 ± 0,33	4,98 ± 0,38	2,67 ± 0,66	1,00 ± 0,09	0,33 ± 0,06	1,29 ± 0,30	0,70 ± 0,13	1,00 ± 0,32	0,88 ± 0,34	1,55 ± 0,40	0,94 ± 0,19
CASP3	TLDA	1,00	0,43	0,88	0,39	0,69	0,34	1,00	0,34	0,56	0,77	1,00	0,70	1,58	1,73	1,00	0,59	1,21	1,43
	RT-PCR	1,00 ± 0,90	0,29 ± 0,23	0,57 ± 0,63	0,21 ± 0,17	0,63 ± 0,53	0,23 ± 0,19	1,00 ± 0,54	0,53 ± 0,41	1,14 ± 0,80	0,91 ± 0,86	1,00 ± 0,18	0,66 ± 0,16	1,95 ± 0,40	1,36 ± 0,42	1,00 ± 0,06	0,53 ± 0,13	1,34 ± 0,38	0,89 ± 0,25
DEDD2	TLDA	1,00	1,08	2,66	1,99	1,14	0,96	1,00	0,82	1,31	1,29	1,00	0,97	1,54	1,88	1,00	1,59	2,94	2,51
	RT-PCR	1,00 ± 0,19	0,56 ± 0,28	1,34 ± 0,69	1,52 ± 0,47	1,09 ± 0,21	0,76 ± 0,10	1,00 ± 0,21	0,79 ± 0,21	2,09 ± 0,44	2,16 ± 0,65	1,00 ± 0,13	1,07 ± 0,09	1,86 ± 0,11	1,96 ± 0,08	1,00 ± 0,22	0,95 ± 0,29	2,40 ± 0,68	1,82 ± 0,54
**MCL-1**	TLDA	1,00	1,27	1,13	2,22	0,82	1,39	1,00	1,11	0,93	1,00	1,00	1,89	1,77	1,86	1,00	1,03	1,58	1,37
	RT-PCR	1,00 ± 1,08	1,14 ± 1,13	1,12 ± 0,62	1,36 ± 1,07	1,00 ± 0,96	1,25 ± 1,05	1,00 ± 0,57	0,90 ± 0,80	1,45 ± 0,90	1,03 ± 0,97	1,00 ± 0,19	1,52 ± 0,21	1,12 ± 0,53	1,15 ± 0,15	1,00 ± 0,57	0,86 ± 0,36	1,43 ± 0,67	0,90 ± 0,35
NALP1	TLDA	1,00	0,18	4,90	2,38	0,79	-	1,00	1,04	9,51	15,6	1,00	1,14	0,89	1,22	1,00	1,94	1,67	1,75
	RT-PCR	1,00 ± 0,65	0,81 ± 0,61	2,48 ± 1,77	1,83 ± 1,59	2,07 ± 2,17	1,75 ± 1,71	1,00 ± 0,90	0,44 ± 0,29	17,3 ± 11,8	7,90 ± 5,27	1,00 ± 0,29	0,84 ± 0,07	0,85 ± 0,18	0,68 ± 0,07	1,00 ± 0,23	1,43 ± 0,29	0,86 ± 0,42	0,98 ± 0,60
**PMAIP1 = NOXA**	TLDA	1,00	0,45	2,70	1,27	0,84	0,48	1,00	0,25	1,92	1,22	1,00	0,93	2,41	2,04	1,00	1,84	7,98	13,9
	RT-PCR	1,00 ± 0,30	0,28 ± 0,08	3,81 ± 0,56	1,51 ± 0,26	0,87 ± 0,87	0,42 ± 0,42	1,00 ± 0,21	0,54 ± 0,21	3,41 ± 1,12	2,57 ± 0,70	1,00 ± 0,26	0,62 ± 0,08	2,47 ± 0,54	1,46 ± 0,50	1,00 ± 0,32	0,57 ± 0,09	8,45 ± 6,58	5,36 ± 3,72
TRADD	TLDA	1,00	0,53	1,68	0,97	0,59	0,77	1,00	1,53	0,54	1,37	1,00	1,24	0,71	1,07	1,00	1,10	0,57	0,93
	RT-PCR	1,00 ± 0,23	0,81 ± 0,25	1,29 ± 0,24	0,88 ± 0,25	1,26 ± 0,48	1,01 ± 0,26	1,00 ± 0,25	1,42 ± 0,38	1,17 ± 0,61	1,04 ± 0,40	1,00 ± 0,11	1,06 ± 0,21	0,89 ± 0,08	0,94 ± 0,04	1,00 ± 0,11	1,10 ± 0,10	0,86 ± 0,16	0,86 ± 0,08

Supplementary table 3
